# Penguins exploit tidal currents for efficient navigation and opportunistic foraging

**DOI:** 10.1371/journal.pbio.3002981

**Published:** 2025-07-17

**Authors:** Richard M. Gunner, Flavio Quintana, Mariano H. Tonini, Mark D. Holton, Ken Yoda, Margaret C. Crofoot, Rory P. Wilson

**Affiliations:** 1 Department for the Ecology of Animal Societies, Max Planck Institute of Animal Behaviour, Konstanz, Germany; 2 Instituto de Biología de Organismos Marinos (IBIOMAR), CONICET, Puerto Madryn, Argentina; 3 Instituto de Investigaciones en Biodiversidad y Medioambiente (INIBIOMA-UNCO, CONICET), San Carlos de Bariloche, Argentina; 4 Swansea Lab for Animal Movement, College of Science, Swansea University, Swansea, United Kingdom; 5 Graduate School of Environmental Studies, Nagoya University, Nagoya, Japan; 6 Department of Biology, University of Konstanz, Konstanz, Germany; 7 Smithsonian Tropical Research Institute, Balboa, Panama; Queen Mary University of London, UNITED KINGDOM OF GREAT BRITAIN AND NORTHERN IRELAND

## Abstract

Animals navigating in fluid environments often face forces from wind or water currents that challenge travel efficiency and route accuracy. We investigated how 27 Magellanic penguins (*Spheniscus magellanicus*) adapt their navigation strategies to return to their colony amid regional tidal ocean currents. Using GPS-enhanced dead-reckoning loggers and high-resolution ocean current data, we reconstructed penguin travel vectors during foraging trips to assess their responses to variable currents during their colony-bound movements. By integrating estimates of energy costs and prey pursuits, we found that birds balanced direct navigation with current-driven drift: in calm currents, they maintained precise line-of-sight headings to their colony. In stronger currents, they aligned their return with lateral flows, which increased travel distance, but at reduced energy costs, and provided them with increased foraging opportunities. Since the lateral tidal currents always reversed direction over the course of return paths, the penguins’ return paths were consistently S-shaped but still resulted in the birds returning efficiently to their colonies. These findings suggest that Magellanic penguins can sense current drift and use it to enhance energy efficiency by maintaining overall directional accuracy while capitalizing on foraging opportunities.

## Abstract 2

How are magellanic penguins able to navigate tidal currents while balancing energy efficiency and foraging opportunities? This study reveals how they adaptively make S-shaped return paths to their colony, optimizing travel in complex oceanic flows.

## Introduction

Animals moving within water or air are subject to external fluid forces in the form of currents that can affect their locomotion, energy expenditure, and ability to navigate (see [1] for review). These forces lead animals to drift away from heading-based trajectories, which poses navigational challenges for any extensive movements, such as during migration and foraging [1–5]. There is thus strong selective pressure for animals to detect and respond effectively to these environmental vectors [6,7]. Several species have evolved strategies to mitigate the impact of currents by, e.g., timing movements to coincide with favorable conditions [8,9], adjusting speed [10,11], or altering headings to compensate for drift [12–15]. For instance, young frigatebirds have been shown to develop drift-compensation strategies over time [16]. Although marine animal navigation has been extensively investigated, the specific corrective mechanisms that enable these animals to maintain their course in the absence of visual landmarks or seabed references remain insufficiently characterized [17]. A standard approach to assessing the effect of drift on animals is to study movements during goal-orientated traveling because the detrimental effects of fluid flow vectors can be examined directly. Such work shows, for example, that sea turtles have appreciable track tortuosity during migrations to their breeding islands [5]. Similar deviations from supposed optimal (in terms of distance and time) trajectories have been observed in birds [18–20], specifically, that these animals have suboptimal trajectories based on extra distance and prolonged travel time due to drift-induced effects of currents. Although previous studies have evaluated animal travel vectors relative to fluid and ground movement, our understanding of how animals maintain a goal-oriented trajectory under environmental drift has been limited by the lack of high-resolution, simultaneous data on animal headings, external fluid vectors, and travel speed.

We used GPS-enabled dead-reckoning on breeding Magellanic Penguins *Spheniscus magellanicus*, central-place foragers operating out of sight of land during the breeding season [21–24], to study their abilities to return to their nests after foraging while exposed to considerable tide-driven current drift [25]. Dead-reckoning integrates compass-derived headings and modeled swimming speeds to reconstruct detailed movement paths of the penguins [26]. This approach provided explicit information on how penguins adjusted their navigation strategies in response to variable ocean current conditions. By integrating estimated travel vectors (pre-current integration) with a hydrodynamic model of ocean currents, we quantified the complexity of their navigational challenges and reconstructed their responses to both minimal currents (slack water) and strong opposing flows. Additionally, we estimated energy expenditure using established power-speed relationships and analyzed dive profiles to assess prey pursuit behavior during their return journeys. These methods allowed us to explore how penguins balance efficient navigation with opportunistic foraging in a dynamic marine environment.

Our objectives were to: (1) investigate whether the penguins showed evidence of sensing current drift in the absence of landmarks; (2) assess the penguins’ ability to orient toward their colony; (3) examine how they changed movement strategy when exposed to substantial currents; and (4) evaluate the energetic costs and distances traveled as a consequence of their navigation strategies.

## Materials and methods

### Study site, subjects, instrumentation and attachment

Fieldwork was conducted between 22 November and 1 December 2019 at the San Lorenzo Magellanic penguin colony, Peninsula Valdés, Argentina (42.08° S, 63.86° W). We selected 27 adult Magellanic penguins (*Spheniscus magellanicus*) brooding small chicks for this study. Each penguin was equipped with a GPS logger (AxyTrek, Technosmart, Italy) and a Daily Diary (DD) logger [27]. The GPS units recorded positions at 1 Hz, while the DD loggers recorded tri-axial acceleration at 40 Hz and tri-axial magnetometry at 13 Hz, and pressure (indicating depth) at 4 Hz. Both devices were housed in hydrodynamic casings designed to minimize drag [28]. Combined, the devices weigh 85.7 g, which corresponds to approximately 2.1% of an average 4-kg bird, with published body masses typically ranging from 3.5 to 4.5 kg [24]. Devices were secured on the dorsal midline with Tesa tape [29] in a process taking less than 5 min. Penguins completed a single foraging trip before recapture.

Ethical approval for the research was granted by Swansea University’s Ethics Committee (SU-Ethics-Student: 260919/1894) and the Animal Welfare and Ethical Review Body (AWERB approval: IP-1819-30). Fieldwork permits were authorized by the Conservation Agency of Chubut Province (Disp N° 047/19-SsCyAP; No. 060/19-DFyFS-MP). All procedures involving penguin handling were reviewed and approved by the Dirección de Fauna y Flora Silvestre and the Ministerio de Turismo y Áreas Protegidas de la Provincia de Chubut.

### Ocean current model and dead-reckoning

To understand how external water movements might shape the penguins’ goal-oriented navigation efficiency, we simulated ocean currents using the Regional Ocean Modelling System (ROMS) [30], incorporating regional bathymetry and tidal constituents from the TPXO6 global model [31]. Model outputs were harmonically analyzed for primary tidal components, with U and V flow components representing east-west and north-south directions, respectively [25] (S1 Text, S1Fig). The model output covered 50,500 km² at 1 km² resolution, spanning 22 November to 2 December 2019.

Penguin horizontal swimming speed was estimated based on their rate of change of depth and body pitch [32] using speed thresholds for surface swimming, low pitch underwater swimming, and high pitch diving (S1 Text). Penguins were dead-reckoned (DR) at 1 Hz resolution using the *Gundogs.Tracks()* function in R [33], with corrections applied based on GPS fixes obtained when the birds surfaced. At 1-s intervals, the U and V current components were bilinearly interpolated at nearest hourly timestamps according to the birds’ DR proximity using the *interpp()* function from the *akima* package in R [34]. Outbound and inbound phases of each foraging trip were identified by cumulative changes in the shortest distance between the penguin and the colony, with the start of a continuous downward gradient marking the homing trajectory (S3 Fig). Additional details on the ocean current model, horizontal swimming speed estimation, and adjustments for pressure sensor drift and tag orientation discrepancies are provided in S1 Text.

### Heading and derivation of traveling vectors

**Fig 1:**
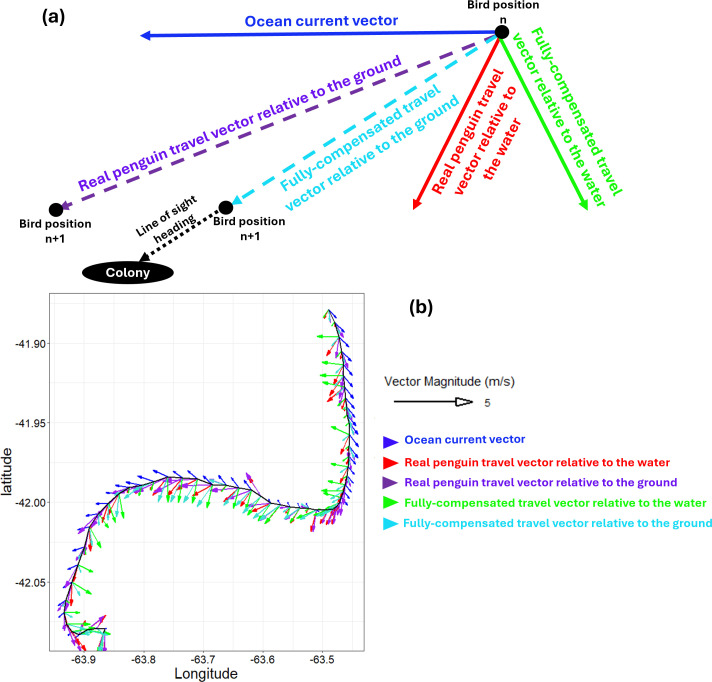
Key vectors in penguin return journey analysis. This diagram illustrates the primary vectors used in analyzing the penguin’s journey back to its colony. In panel **(a)**, the ocean current vector (solid blue) combines with either the real penguin travel vector relative to the water (solid red) or the fully-compensated travel vector relative to the water (solid green) at position ‘*n*.’ These interactions result in the projected real penguin travel vector relative to the ground (dashed purple) or the projected fully-compensated travel vector relative to the ground (dashed cyan) at position ‘*n* + 1’. The dotted black arrow represents the direct line-of-sight path to the colony. The fully-compensated travel vector relative to the ground is aligned with this line-of-sight path unless the penguin’s speed is insufficient to fully counteract the current. Panel **(b)** shows a sample of these vectors recalculated every 5 min along a penguin’s southward return journey, based on its dead-reckoned track. The fully-compensated travel vector relative to the water (solid green) sometimes counteracts the ocean current at angles greater than 90° to help the penguin stay on the most efficient path back to the colony.

To quantify how penguins orient themselves relative to both ocean currents and their target (the colony), we derived five two-dimensional vectors from bird and ocean current headings/speeds (Fig 1):

(i) **The ocean current vector:** This represents the speed and direction of water movement from the ocean current model, with U (east–west) and V (north–south) components.
(ii) **The real penguin travel vector relative to the water (pre-ocean current integration):** This is the penguin’s travel vector as if it were unaffected by currents. It is calculated from the penguin’s heading (from the tag compass [33]) and speed, converted to U and V components. This vector represents the penguin’s movement relative to the surrounding water, prior to GPS dead-reckoning (DR) correction.
(iii) **The real penguin travel vector relative to the ground (post-ocean current integration):** This describes the penguin’s effective movement across the ground, accounting for ocean current influence. It is computed by summing the bird’s vector relative to the water (see ii) and the ocean current vector (see i), providing U and V components of the penguin’s overall ground movement.
(iv) **The fully-compensated travel vector relative to the water:** At each point along the DR track, we calculated the heading which, combined with the penguin’s speed (pre-DR correction and without ocean current integration), would produce a vector as close as possible to a direct ‘line-of-sight’ path back to the colony after integrating with the local ocean current vector. If the penguin’s speed was insufficient to counter strong opposing currents, the ‘next best’ heading was selected to yield a travel vector nearest to the desired line-of-sight direction.
(v) **The fully-compensated travel vector relative to the ground:** Calculated as the sum of U and V components from (i) and (iv). In most cases, this vector closely matched the line-of-sight heading.

Conversion between U and V components and speed and direction is detailed in S2 Text.

### Ease of transport

To evaluate the energetic consequences of real versus fully-compensated travel vectors, we used an inverse of the cost of transport (COT) [cf. 35], defined as ‘ease of transport’ (the number of meters travelled per joule of energy (m/J)). This metric—analogous to fuel efficiency (‘miles per gallon’ or ‘km per litter’) used by the vehicle industry—was used to emphasize how much the pathways utilized by penguins facilitated their progression within their moving oceanic landscape. Power requirements for the penguins’ chosen speed were calculated using the formula from Luna-Jorquera and Culik [36], relating mass-specific power to swim speed for the morphologically and physiologically similar congeneric Humboldt penguins (*Spheniscus humboldti*) [33].


$$Power=2.954\cdot s^{3}-6.354\cdot s^{2}+5.818\cdot s+5.9$$ (1)

where s is the horizontal swimming speed (m/s) and Power is in W/kg (S1 Text). These values were then multiplied by 4 kg (the average weight of Magellanic penguins [24]) to convert to energy usage per penguin per second (J/s).

At each point along the DR track during the penguins’ inbound paths, coordinates were advanced based on the speed and direction of either the real penguin travel vector relative to the ground or the fully-compensated travel vector relative to the ground. For each vector, we then computed two variants of ease of transport (cf. Box 1):

**Box 1. Conceptual overview of ease transport variants.**

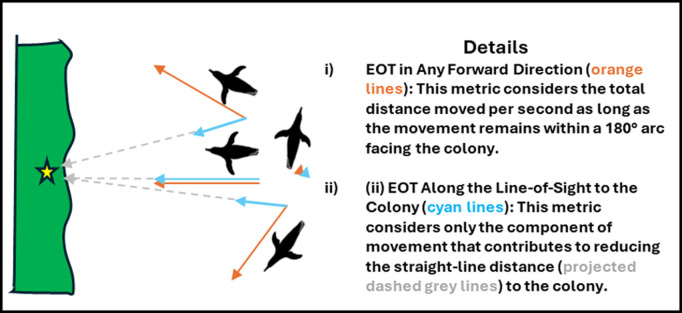

(i) **The ‘ease of transport’ in any direction that reduces the distance to the colony:** This metric represents the energy efficiency of movement in any forward direction per unit power cost. It is calculated as the distance moved per second divided by the power cost (i.e., the distance between bird positions *n* and *n* + 1 in Fig 1). Only movements within a 180° arc facing the colony were included; movements away from the colony were assigned an ease of transport value of zero.
(ii) **The ‘ease of transport’ along the line-of-sight to the colony:** This metric represents energy efficiency of movement directly towards the colony, calculated as the distance covered along the line-of-sight trajectory to the colony per second, divided by power cost (i.e., the change in the line-of-sight distance between bird positions *n* and *n* + 1 in Fig 1). Negative ease of transport values, arising when birds moved away from the colony, were set to zero.

### Agent-based model

To compare real trajectories versus a naive heading approach, we simulated penguin return journeys under a theoretical scenario where each penguin (agent) maintained a fixed initial line-of-sight heading toward the colony, never adjusting for cumulative current drift. Each agent used the real penguin’s speed data, iteratively applying its initial heading with time- and space-matched current vectors until either reaching the colony or failing to return to the colony’s longitude before the real penguin.

### Dive depth and prey pursuits

We also explored whether foraging activity might influence heading decisions by investigating diving behavior during return journeys. We determined maximum dive depth (>0.3 m) and computed a prey pursuit index during dives based on ‘wiggles’ (undulations) in the depth profile following Simeone and Wilson [37]. Here, a single wiggle during which the penguin vertical velocity exceeded 0.3 m/s over ≥1 s was considered to represent a prey capture [37].

### Statistical analysis

#### Data binning and normalization.

To address unequal sample sizes between penguins, we binned variables of interest, with counts normalized to proportions per penguin and averaged to yield grand mean relative frequencies. For analyses by distance travelled during return journeys, means were calculated per bin for each penguin and then averaged (S3 Text).

#### Bootstrapped Kolmogorov–Smirnov (KS) test.

A bootstrapped KS test was used to compare penguin heading deviations between outbound and inbound journeys under varying current strengths (‘Slack’ < 0.3 m/s versus ‘Appreciable’ ≥ 0.3 m/s), accounting for sample variability and intra-individual effects (S3 Text). Approximately 30% of current data fell under the ‘Slack’ threshold, which was defined as less than 0.3 m/s—about half the mean current strength across all tracks. This threshold is below the penguins’ surface swimming speed, making it a logical boundary relative to their lowest observed travel speeds.

#### Generalized additive models (GAMs).

We employed GAMs (*mgcv* package in R) to investigate non-linear relationships between behavioral and environmental factors (see S3 Text for full details):

1. **Ease of transport**: GAMs modelled the ease of transport relative to distance travelled, comparing real and fully-compensated travel vectors, with random smooth effects to account for individual variation across the return distance (S3 Text).
2. **Heading deviation**: This GAM assessed factors influencing the deviation of the real bird travel vector heading from the line-of-sight to the colony. It included random smooth effects for individual variation across the return distance, with separate smooth terms for current speed, angular deviation between penguin and ocean current headings, resultant travel speeds (post- versus pre-current integration), maximum dive depth, and rate of prey pursuits (S3 Text and S4 Fig).

## Results

### Penguin movement patterns

**Fig 2:**
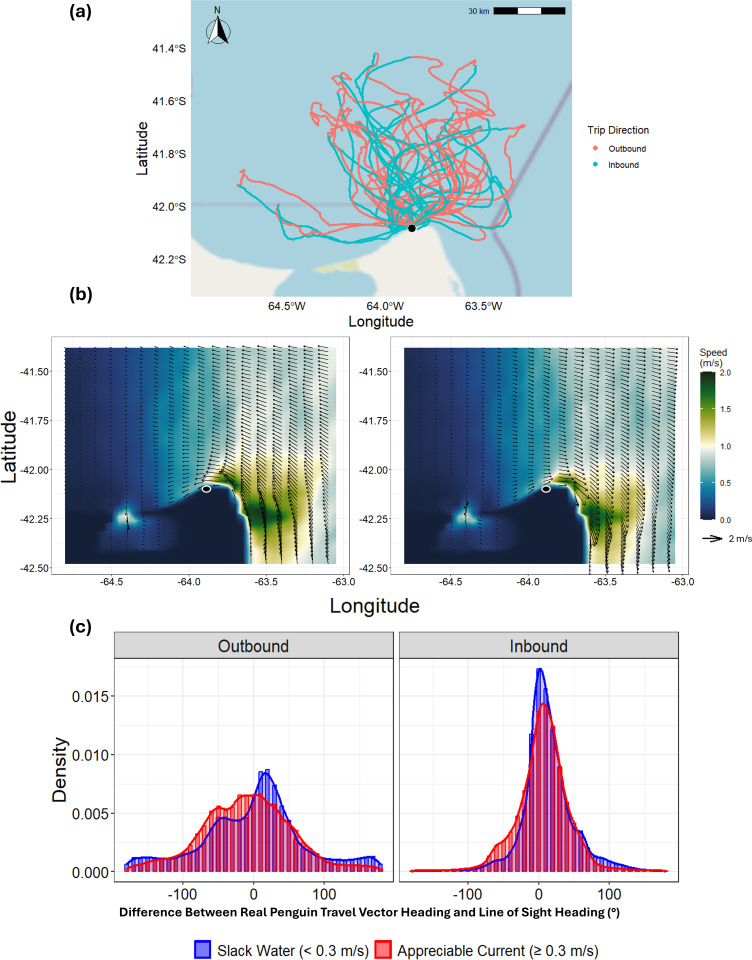
Overview of penguin tracks, regional currents, and heading distributions. **(a)** The GPS-corrected dead-reckoned tracks of 27 penguins at sea, colored to distinguish between the outbound (red) and inbound (blue) phases of their foraging trips. The base map was constructed using OpenStreetMap (OSM) tiles—licensed under the Open Data Commons ODbL. **(b)** A ‘snapshot’ of the current conditions within the grid area, showing peak tidal strength during a single day as a function of the tidal cycle, including the outgoing (‘ebb’ – left panel) and incoming (‘flood’ – right panel) tides. Penguins are often subjected to strong cross-currents during these phases. In both **(a and b)**, the colony location is marked by a small black filled circle with a white outline. **(c)** Distribution of heading differences between the penguin’s travel vector heading (relative to water) and the direct line-of-sight heading, shown for Slack Water (<0.3 m/s, blue) and Appreciable Current (≥0.3 m/s, red), and for outbound versus inbound phases. A heading difference of 0° indicates direct movement toward (inbound) or away from (outbound) the colony, whereas ±90° is perpendicular movement. In both ‘Slack Water’ (<0.3 m/s, blue) and ‘Appreciable Current’ (≥0.3 m/s, red) conditions, penguins align more closely with the line-of-sight heading during the return (inbound) phase, suggesting a more focused return to the colony compared to the departure (outbound) phase. The data underlying this figure can be found in https://doi.org/10.6084/m9.figshare.28517873.

To characterize the overall foraging trajectories, we first examined the paths and durations of the penguins’ trips (Fig 2). Twenty three penguins exhibited ‘looping pathways’ [cf. 38], heading approximately northward to forage and returning southward to their colony. Return tracks often displayed an S-shape (Fig 2a), being influenced by the prevailing ocean currents which predominantly ran East–West or West–East depending on the phase of the tidal cycle (Fig 2b). On average (mean ± SD), the return journeys began 50 ± 15 km (range: 20–75 km) from the colony and took 12 ± 4 hrs to complete, whereas outbound journeys lasted 20 ± 9 hrs. Penguins exposed to cross-currents during both the outbound and inbound phases spent approximately equal time navigating eastward and westward currents. During the outbound phase, they spent 50.7% ± 10.5% of the time in eastward currents and 49.3% ± 10.5% in westward currents [(paired *t* test: *t* = −0.366, *p* = 0.717)]. Similarly, during the inbound phase, they spent 49.3% ± 14.6% of the time in eastward currents and 50.7% ± 14.6% in westward currents [(paired *t* test: *t* = 0.237, *p* = 0.814)]. The average current strength across all tracks was similar between the two phases, averaging 0.59 ± 0.23 m/s during the outbound phase and 0.60 ± 0.20 m/s (max 2.0 m/s) during the inbound phase (cf. S4 Fig). These represent a significant fraction (mean 29%, max 95%) of normal penguin travelling speed of *ca*. 2.1 m/s [39,40] and should have a corresponding influence on their trajectories. Birds landed on the coast at minimal distance from their departure point at the colony (0.17 ± 0.18 km, range: 0.01–0.72 km), with 85% of birds returning within 0.3 km. Eight of 27 penguins returned at night (10 PM to 4 AM).

### Influence of currents on heading deviation

To assess how penguins orient to the colony, we compared their travel vector heading (relative to the water) with the line-of-sight heading during both outbound and inbound phases, under ‘slack water’ and ‘appreciable current’ conditions (Fig 2c). A bootstrapped KS test showed that during the inbound phase, penguin travel vector headings relative to the water were more tightly distributed around their line-of-sight heading compared to the outbound phase (D = 0.232, 95% CI: 0.228–0.241; Fig 2c). The variability of heading strategies was significantly influenced by current strength and trip phase (Table 1). During inbound movement, on average, penguins directed themselves within 25° of the line-of-sight heading 60% of the time and within 45° 80% of the time, rarely deviating more than 90° regardless of current conditions (Figs 2c and S2). Although these results illustrate a strong alignment with the colony’s direction, a separate analysis of the birds’ absolute headings (S5 Fig) reveals a broadly bimodal distribution, indicating that penguins do not adhere to a single compass heading per se.

**Table 1:** Pairwise Kolmogorov–Smirnov (KS) test results for penguin heading distributions relative to the line-of-sight heading. Comparisons are made for real penguin vector headings relative to the water across different ocean current conditions (‘Slack Water’ < 0.3 m/s and ‘Appreciable Current’ ≥ 0.3 m/s) and trip phases (Outbound vs. Inbound). The *D* statistic represents the maximum difference between the cumulative distributions of the two compared groups, with corresponding *p*-values indicating significance.

Comparison	*D*-statistic	*p*-value
Appreciable Current (Outbound) vs. Slack Water (Outbound)	0.0386	<.001
Appreciable Current (Outbound) vs. Appreciable Current (Inbound)	0.2136	<.001
Appreciable Current (Outbound) vs. Slack Water (Inbound)	0.2679	<.001
Slack Water (Outbound) vs. Appreciable Current (Inbound)	0.1902	<.001
Slack Water (Outbound) vs. Slack Water (Inbound)	0.2424	<.001
Appreciable Current (Inbound) vs. Slack Water (Inbound)	0.0575	<.001

To understand how ocean currents affect penguin navigation during the inbound phase of their foraging trips, we analyzed the relationship between penguin travel vector headings and their line-of-sight direction to the colony in relation to ocean current direction.

Before accounting for ocean current effects, the real penguin travel vector headings relative to the water (represented by the red arrow in Fig 1) sometimes required the penguins to swim against the ocean current to maintain their line-of-sight heading (dotted black arrow in Fig 1) (see S6a Fig). When ocean current vectors were included, the resultant penguin travel vector headings relative to the ground (purple arrow in Fig 1) showed more pronounced deviations from this direct line-of-sight path (see S6b Fig).

In contrast, theoretically fully-compensated travel vector headings relative to the water, calculated before integrating ocean current effects (green arrow in Fig 1), would often require the penguins to swim at angles greater than 90° from the ocean current direction to achieve a resultant travel vector heading leading directly back to the colony (cyan arrow in Fig 1) (see S6c and S6d Fig).

**Fig 3:**
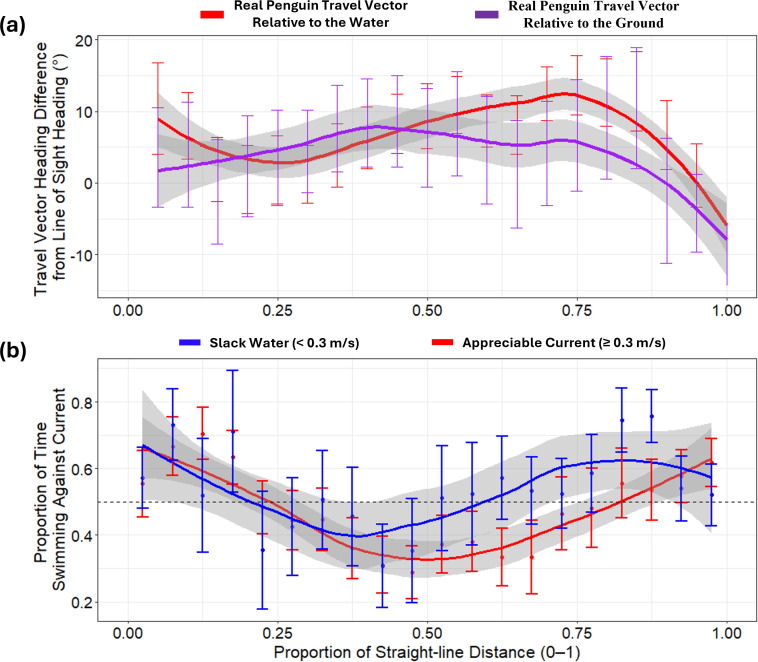
Variation in penguin headings relative to line-of-sight and proportion of time spent swimming against ocean currents during the return journey. **(a)** Mean (±1 SE) angular difference between real penguin travel vector headings and the line-of-sight direction to the colony. Headings are shown relative to the water (before accounting for ocean currents; red) and relative to the ground (after accounting for ocean currents; purple), with fitted loess smooth lines. For a complementary measure that factors in speed (the “Deviation Index ”), seeS7 Fig. . . **(b)** Mean (±1 SE) proportion of time penguins spent swimming against the ocean current. Blue represents periods with “slack water” (<0.3 m/s), while red indicates periods with “appreciable current” (≥0.3 m/s). Time spent swimming against the current is defined as instances where heading angles exceeded 90° in absolute terms. The dashed horizontal line at *y* = 0.5 marks the 50% threshold, indicating equal time spent swimming with and against the current. In both panels, the *x*-axis is divided into 0.05 increments of straight-line distance travelled. The data underlying this figure can be found in https://doi.org/10.6084/m9.figshare.28517873.

We also examined how heading strategies varied along the journey using a distance-based perspective (Fig 3). Variation in penguin travel vector headings around the line-of-sight heading differed notably among individuals and changed non-linearly with distance to the colony (Fig 3a). Penguins showed a general trend toward more goal-oriented behavior at the start and near the end of their return journeys, with greater deviations in the middle. This pattern coincided with variations in the proportion of time spent swimming against ocean currents, especially under appreciable current strength (Fig 3b). Specifically, the penguins’ travel vector headings relative to the water often deviated from the line-of-sight—indicating active swimming at angles to counteract stronger currents—so that their net travel vector headings relative to the ground remained more closely aligned with the colony’s line-of-sight trajectory (S7 Fig).

### Ease of transport

**Fig 4:**
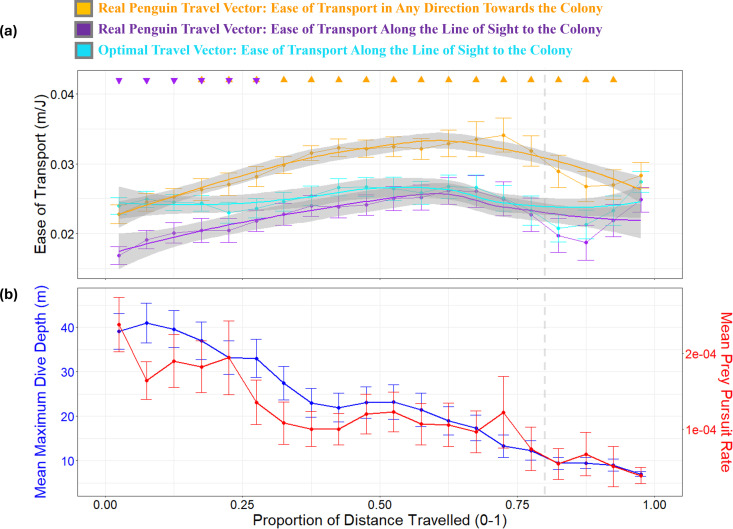
Penguin ease of transport for real vs. fully-compensated travel vectors relative to the ground during the return journey with respect to depth use and prey acquisition. **(a)** Mean ease of e of transport (±1 SE, in m/J) is shown across the proportion of the return distance to the colony for the real penguin travel vector in any direction towards the colony (orange), the real penguin travel vector along the line-of-sight to the colony (purple), and the fully-compensated travel vector (cyan). Higher values indicate greater energy efficiency of movement. Significant pairwise differences (*p* < 0.05) between the real and fully-compensated vectors are marked with solid triangles at 0.05 intervals of the return journey, based on GAM-predicted ease of transport values, which account for individual variability and smooth trends over distance (see e S3 Text for details details). Upward-facing triangles indicate that the real travel vector (orange or purple) had significantly higher ease of transport than the fully-compensated vector (cyan), while downward-facing triangles indicate the opposite. The absence of a triangle indicates no significant difference at that interval. **(b)** Mean maximum dive depth (±1 SE, in m) and mean prey pursuit rate (±1 SESE, pursuits s^−^¹), plotted at 0.05 intervals of the return journey’s proportion of distance travelled. Note that the overall depth decreases over time showing that birds allocate increasing time to horizontal travel. Dive depths decrease to less than 10 m when *ca*. Eighty percent of the journey is complete (marked by the dashed grey vertical line). This pattern corresponds with substantive changes in the ease of transport and a corresponding reduction in prey pursuit. Importantly, this reduction in dive depth over time was not influenced by the surrounding seafloor depth (S8 Fig*). The* data underlying this figure can be found in https://doi.org/10.6084/m9.figshare.28517873.

We next investigated whether real versus fully-compensated travel vector heading choices translate into differences in energy efficiency (Fig 4a). Penguins achieved higher ease of transport values by moving generally, but not directly, toward the colony, following their real travel vector headings relative to the ground, compared to the theoretical fully-compensated travel vector scenario (Fig 4a). For movement along the line-of-sight path, ease of transport was generally slightly lower at the start of the return journey, aligning more closely with the fully-compensated travel vectors in the middle section, a pattern that coincided with increased time spent swimming with ocean currents during this part of the journey (Fig 4b).

(i) **The ease of transport in in any direction that reduces the distance to the colony:** The GAM analysis indicated that the theoretical fully-compensated travel vectors had a significantly lower ease of transport than the real penguin travel vectors across the return journey (Estimate = −0.0047, SE = 0.0005, *p* < 0.001; Fig 4a, S3 Text and S1 Table). Pairwise comparisons showed that this significance spanned the majority of the return journey (Fig 4a).
(ii) **The ease of transport along the line-of-sight to the colony:** The GAM analysis indicated that the theoretical fully-compensated travel vectors had a slightly higher ease of transport along the line-of-sight path than the real penguin travel vectors (Estimate = 0.0023, SE = 0.0005, *p* < 0.001; S3 Text and S1 Table). Pairwise comparisons revealed that this significant difference was limited to the early phases of the return journey (Fig 4a).

### Agent-based model

To test whether ongoing heading adjustments were necessary given the region’s alternating east–west currents, we modeled a “what if” scenario in which each bird maintained a fixed heading with no drift correction. Many simulated birds ended up returning to land far from their breeding colony (S9 Fig).

### Heading deviation from line-of-sight

Penguins continued to dive to depths exceeding 10 m—presumably capturing prey [37]—for up to approximately 80% of their return journey (Figs 4b, S8, and S10). After this point, dive depths decreased steadily, coinciding with fewer prey captures (Fig 4b) and more consistent, directed travel toward the colony (Figs 3a and S7). The GAM revealed that deviation of bird vector headings relative to the water from the line-of-sight heading was significantly influenced by individual differences, trip progression, and environmental factors. Penguins adjusted their navigation strategies along the return journey, with significant effects observed for the angular difference from the ocean current, maximum dive depth, and resultant speed after current integration. Additionally, prey pursuits influenced heading deviation, and a complex interaction among ocean current speed, alignment with ocean current direction, and resultant bird vector speed differences also impacted heading deviation (S3 Text). Notably, higher prey pursuit rates were positively associated with heading deviation, indicating that birds engaged in more foraging activity tended to deviate further from the colony line-of-sight (S11 Fig). Furthermore, birds exhibited more variable headings when aligning their travel vectors with stronger currents, ultimately yielding higher resultant travel speeds (S11 Fig).

## Discussion

### Adaptation to current conditions and goal-oriented navigation

Magellanic penguins must navigate efficiently over long distances to return to their nests and feed their chicks. Our findings show that they often orient themselves toward the colony by following a general line-of-sight heading (Figs 2, S6 and S12), especially under calm ocean current conditions (Fig 2c). These observations point to a robust, goal-oriented navigation capacity that remains effective even without visible landmarks [41–43]. Given the penguin’s low profile and minimal visibility at the sea surface, visual contact with the colony is unlikely for most of the journey, implying reliance on alternative sensory mechanisms. Indeed, seabirds flexibly use a wide range of cues—including olfaction, magnetoreception, infrasound, sun/star compasses—to orient over medium and long distances [44–48]. In Procellariiform seabirds, olfaction is well documented; for instance, anosmic individuals show impaired homing orientation but unchanged foraging behavior [44], suggesting that smell may be especially critical during homeward phases. Although penguins are not airborne foragers, they also possess a functional sense of smell and may detect odors (e.g., dimethyl sulphide) or colony scents to navigate over open ocean and near land [e.g., 49].

Notably, because Magellanic penguins in our study rarely reach the seabed (S8 Fig), they are unlikely to use benthic cues for perceiving current drift. This contrasts with findings in yellow-eyed penguins [50,51], where seabed features can promote consistent foraging routes. Instead, Magellanic penguins likely rely on other sensory cues to navigate in dynamic marine environments. While geomagnetic cues remain a likely orientation mechanism for many birds [52], mechanoreception—directly sensing water flow or pressure changes—is another possibility [cf. 53]. However, our results indicate that Magellanic penguins may perceive currents indirectly, noticing discrepancies between their intended path (e.g., *via* compass orientation) and actual displacement over ground, then adjusting accordingly. This interpretation is supported by the differences in heading distributions under slack versus stronger currents (Fig 2c). While penguins still aim broadly toward the colony under strong currents (mean heading remains similar), they exhibit a more dispersed heading distribution, potentially reflecting repeated or fine-scale corrections to compensate for the drift. Such behavior is consistent with effective navigation even when out of sight of land.

We also examined how heading strategies varied across the return journey using a distance-based perspective (Fig 3). Penguins generally showed more goal-oriented behavior (relative to the ground) at the beginning and end of their trips, with greater deviations in the middle (Fig 3a). This pattern coincided with the penguins swimming at angles ≥90° to the ocean current, reflecting active drift compensation (Fig 3b). Specifically, while their water-referenced travel vector headings often deviated from the line-of-sight (to counteract lateral currents), the ground-referenced headings generally remained more closely aligned with the colony’s line-of-sight trajectory. In other words, by angling themselves against the current, the penguins minimized net drift and ultimately maintained a more direct path home. This early precision reduces error accumulation over long distances—important because even small deviations at the start of a long return journey could otherwise compound into large errors. In contrast, mid-journey deviations appear when birds are influenced by strong currents although these are subsequently partially corrected as they near the colony. Closer to the colony, penguins again show a strong tendency to follow the line-of-sight, potentially aided by visual cues in the final approach—similar to behaviors observed in shearwaters [54]. However, since approximately 30% of penguins returned at night, visual cues alone may not be essential for homing [cf. 54] although lack of visual cues at this time this may explain the slight deviation from proper line of sight over the last few meters (Fig 3a). Nevertheless, as birds near their nesting site, any deviation from a direct trajectory becomes increasingly costly, reinforcing the importance of precise navigation during these final stages of the journey. Impressively, 85% of penguins returned within 0.3 km of their departure points, matching the accuracy reported by Quintana and colleagues [48]. To illustrate, for an average 50 km journey, a 300 m deviation represents a 99.4% direct return efficiency. Magellanic penguins exhibit strong parallels in their marine and terrestrial navigation strategies, suggesting they may rely on similar orientation mechanisms in both environments. Quintana and colleagues [48] observed that upon returning from the sea, penguins initially traverse a steep, featureless pebble beach with minimal landmarks, following direct paths toward their nests—akin to the line-of-sight headings we observed at sea. A similar pattern occurs in the final 6–7 m before reaching the nest, while mid-journey deviations were more pronounced, likely due to obstacles such as dense vegetation.

### Flow-assisted navigation and adaptive heading adjustment for energy efficiency

The tidal cycles in the San Matías Gulf expose penguins to alternating east-west currents in a semi-diurnal cycle (approximately 12.4 hrs, S1 Fig), causing lateral drift but also presenting opportunities for current-assisted movement (Figs 2, S2, S13 and S14). Although there are some differences in current speeds—for example, during inbound journeys, eastward currents averaged 0.69 ± 0.24 m/s compared to 0.49 ± 0.18 m/s for westward currents—the tidal cycle ensures that penguins encounter opposing currents in roughly equal measure (S4 Fig). This balance in exposure, even if the speeds are not identical, allows the overall current regime to partially cancel out lateral drift over the course of the journey, reducing the need for continuous active correction by the birds. This mean, that birds in this region can facilitate for lateral drift because it can later be corrected by reversed currents (e.g., Figs S13 and S14). Specifically, they adjust their headings to avoid opposing currents and take advantage of favorable flows, when possible, sometimes angling their path relative to the line-of-sight to maintain forward progress while conserving energy (e.g., Figs 3, S13, S14). This flow-assisted movement behavior [1] is evident in the ease of transport values: penguins’ real travel vectors achieved higher efficiency relative to the ground than the theoretical fully-compensated vectors (which prioritize the most direct movement back to the colony) with birds nonetheless decreasing the distance between themselves and the colony (orange line in Fig 4). By harnessing current strength (Figs 3, S2, S6 and S12), penguins enhanced their travel efficiency in a manner similar to birds using tailwinds during migration [7,55].

Swimming with the current during mid-journey stages reduces drag-related energy costs and conserves energy compared to swimming directly against currents [56–58], which is crucial given the exponential increase in power costs at higher swimming speeds (see (Eq 1)). We suggest that this strategy also allows penguins to search for prey opportunistically [59] because dive data reveal frequent deep dives during the return trip where penguins captured prey (Fig 4b, S10 and S14), even though surface swimming typically offers the most energy-efficient travel for air-breathing marine animals [60,61]. Although these prey pursuits are associated with deviations from a direct line-of-sight heading, the observed heading changes may primarily reflect an opportunistic response to encountering prey rather than a calculated trade-off between energy conservation and foraging gain. Notably, the ease of transport along the line-of-sight to the colony was only slightly higher overall in the theoretical fully-compensated scenario (cyan versus purple lines in Fig 4a), with penguins’ real movements achieving comparable efficiency despite deviating from a direct line-of-sight heading (Figs S6b and S12). In other words, by adjusting their headings to take advantage of favorable currents, penguins can maintain efficient progress toward their goal, even if their path deviates from the most direct route. Whether penguins rely solely on directional cues or also possess an exact distance-based cognitive map remains uncertain and warrants further investigation.

### Navigation strategies and environmental complexity

The variable tidal flows in the San Matías Gulf present a dynamic and challenging environment for navigation. Given the alternating eastward and westward currents, one might expect penguins to maintain a constant, line-of-sight heading to the colony; however, our agent-based model (S9 Fig) shows that a purely fixed heading often causes birds to come ashore far from their nest site, underscoring the need for ongoing drift corrections. In reality, penguins follow a more flexible, non-linear navigation strategy (Figs 3 and 4). This strategy is complex, as shown by our GAM analysis, which revealed that deviations in bird vector headings relative to the water from the line-of-sight heading were significantly influenced by multiple factors, including individual differences, current speed, alignment with the current, opportunities for prey pursuits, and dive behavior (S3 Text, S2 Table and S11 Fig).

This variety of influences underscores the intricate nature of penguin navigation in an ever-changing foraging environment, and indicates that their movement strategies are complex, balancing straight-line navigation with current-driven drift over current regimes, time, space, and in response to prey availability. This adaptive strategy echoes behaviors observed in other penguin studies. For example, in a manner similar to king penguins exploiting mesoscale eddies to expedite travel to foraging grounds [62], our Magellanic penguins exploited tidal currents to reduce transit effort. In the Beagle Channel, Magellanic penguins likewise were shown to take advantage of current flow to increase their speed without increasing their energetic costs, suggesting this behavior is common in the species [59]. However, unlike king penguins, that ignore currents when rushing back to the colony [62], Magellanic penguins in our study allowed currents to deflect them into an S-shaped route on the return, taking advantage of reversible tidal flows. We suggest that similar complexities may occur in animal navigation challenges elsewhere and that these may account for departures from expected routes [5].

## Supporting information

S1 Text Numerical ocean current model, dead-reckoning speed estimates, and GPS-corrected dead-reckoning procedure. (DOCX)

S2 Text Conversions between U and V components (m/s) and heading (°) and speed (m/s). (DOCX)

S3 Text Statistical methods expanded. (DOCX)

S1 Table Summary of GAM results for ease of transport models: (i) The real penguin travel vector relative to the ground. **(ii)** The fully-compensated travel vector relative to the ground.
(PDF)

S2 Table Summary of GAM results for deviation of penguin heading from line-of-sight heading. (PDF)

S1 Fig Time series of elevation and ocean current components highlighting cross-current variations. The top panel shows the variation in elevation and the U (east–west) and V (north–south) components of ocean currents in the study region over a 40-day period. The bottom panel presents a zoomed-in view of a 10-day section focusing on the phases of the U and V current components. The plot demonstrates periods dominated by cross-currents, particularly highlighting the U component’s influence on ocean dynamics. The data underlying this figure can be found in https://doi.org/10.6084/m9.figshare.28517873.
(TIF)

S2 Fig Penguin heading distributions during outbound and inbound phases of foraging trips under different current conditions. This plot shows the difference between the penguin’s travel vector heading (relative to the water) and the ocean current direction. The distribution is often bimodal, likely due to encounters with cross-currents. This bimodality is more pronounced during the return phase, suggesting more frequent cross-current navigation. The data underlying this figure can be found in https://doi.org/10.6084/m9.figshare.28517873.
(TIF)

S3 Fig Determining the start of a penguin’s return journey using GPS data and distance to colony. The left plot depicts the shortest straight-line distance from the penguin to the colony over time. The red vertical line indicates the start of the return phase, determined by selecting all data after the first GPS fix that occurred during the beginning of a continuous downward trend in straight-line distance to the colony. The right plot shows the penguin’s movement track (15-min GPS fix intervals shown). The blue section of the track represents the ‘Inbound’ phase, corresponding to the data after the red vertical line in the left plot. The data underlying this figure can be found in https://doi.org/10.6084/m9.figshare.28517873.
(TIF)

S4 Fig Proportion of time spent in eastward versus westward currents and activity types during outbound and inbound trips. **(a)** Shows the proportion of time penguins experienced eastward-facing and westward-facing currents during outbound and inbound trips. For Inbound trips, the mean (±1 SD) current speed was 0.686 ± 0.243 m/s for eastward currents and 0.489 ± 0.183 m/s for westward currents; for Outbound trips, mean speeds were 0.628 ± 0.291 m/s (eastward) and 0.533 ± 0.178 m/s (westward). **(b)** Displays the proportion of time spent in each activity type (resting versus commuting) during outbound and inbound trips. Resting was defined as surface periods ≥60 s and commuting-specific to this figure-as all other times. Boxplot (boxes encompass the 25% to 75% interquartile range, horizontal bars reflect the median and whiskers extend to 1.5 * Interquartile range). Jittered points show each bird’s data point. The data underlying this figure can be found in https://doi.org/10.6084/m9.figshare.28517873.
(TIF)

S5 Fig Distribution of penguin travel vector headings (relative to the water). A circular density plot (polar coordinates) shows the absolute heading (0–360°, binned in degrees from North) for inbound (blue) versus outbound (red) travel phases. Both phases exhibit a broadly bi-modal distribution. The polar axis begins at 0° (North) at the top, increasing counterclockwise to 360°. Density is depicted radially, and overlapping areas indicate where inbound and outbound headings coincide. The data underlying this figure can be found in https://doi.org/10.6084/m9.figshare.28517873.
(TIF)

S6 Fig 2D-binned contour plots of real versus theoretically fully-compensated penguin travel vectors during the return phase of foraging trips. These contour plots compare real and theoretically fully-compensated penguin travel vectors relative to the line-of-sight heading to the colony (*X*-axis) and the ocean current direction (*Y*-axis). **(a)** Real penguin travel vector headings relative to the water generally align with line-of-sight trajectories toward the colony, though some skew is evident. **(b)** When accounting for ocean current effects, the resultant real penguin headings relative to the ground reveal current-assisted trends, roughly indicated within the dashed yellow lines. These headings show increased dispersion around the line-of-sight heading, with more occurrences of nearly opposing headings. **(c)** The theoretically fully-compensated penguin travel vector headings relative to the water, which ideally allow the birds to follow a direct line-of-sight path back to the colony after factoring in ocean current effects (as seen in panel **d**), would often require significant time swimming against the current, especially outside the dashed yellow lines. The data underlying this figure can be found in https://doi.org/10.6084/m9.figshare.28517873.
(TIF)

S7 Fig Deviation index across the return journey. This index represents the lateral/off-axis component of each penguin’s travel relative to the direct line-of-sight heading. The red curve (mean ± SE) is computed using the bird’s original speed (before current integration) multiplied by sin(*Δθ*), where *Δθ* is the angular difference between the bird’s heading relative to the water and the line-of-sight. The purple curve (mean ± SE) uses the resultant (ground-based) speed and heading. Both are plotted against the proportion of straight-line distance (0–1) remaining in the return journey. The data underlying this figure can be found in https://doi.org/10.6084/m9.figshare.28517873.
(TIF)

S8 Fig Bathymetry estimates of the San Matías Gulf. **(a)** Estimated seafloor depths in the region encompassing all penguin foraging journeys. **(b)** Mean maximum dive depth as a percentage of seafloor depth (±1 SE) across proportions of total distance travelled during return trajectories. Percentage values were averaged per 0.05 increments of total distance travelled. Bathymetric map derived from GEBCO_08 data. The data were obtained under GEBCO’s terms of use. Bathymetry is shown as a ‘bathy’ object in R using the marmap package [63]. Depth values (m) in **(a)** are plotted with a custom color ramp to highlight shallower shelf regions. The data underlying this figure can be found in https://doi.org/10.6084/m9.figshare.28517873.
(TIF)

S9 Fig Lateral displacement of penguins relative to departure point during return journeys. This figure illustrates the lateral displacement of penguins from their departure point at the colony during their return journeys. The *y*-axis represents lateral displacement (positive values indicate eastward movement; negative values indicate westward movement), and the *x*-axis represents the proportion of the total distance travelled (from 0 to 1). **(a)** Actual penguin data: Each line depicts an individual penguin’s lateral displacement over time. **(b)** Simulated scenario: Each penguin follows a fixed line-of-sight heading toward the colony without adjusting for current-induced drift effects. For both real and simulated penguins, starting coordinates were used as baselines to calculate absolute lateral displacement. The purple dashed boxes highlight displacement within 15 km of the colony during the final stages of the of the total distance travelled. The data underlying this figure can be found in https://doi.org/10.6084/m9.figshare.28517873.
(TIF)

S10 Fig Prey pursuits and dive depth versus dive depth. **(a)** The cumulative number of prey pursuits as a function of 3D distance travelled across each bird’s dead-reckoned path. Undulations in the dive profile were used as a proxy for prey pursuits, following methods outlined in Simeone and Wilson [37]. **(b)** The plots are faceted based on the number of prey pursuits per dive, illustrating how dive behaviour varies with foraging activity. The data underlying this figure can be found in https://doi.org/10.6084/m9.figshare.28517873.
(TIF)

S11 Fig Three-dimensional plots generated using ‘vis.gam’ provided insights into how pairs of predictors interact to influence penguins’ deviation from their intended line-of-sight direction (cf. S2 Table). **(a and b)** Due to the nature of cross-currents in the region, penguins tend to align their movement with ocean current flow vectors. This alignment results in greater deviation from their line-of-sight trajectory, particularly at higher current speeds. However, this alignment also enables penguins to travel greater distances per unit time by increasing their resultant speeds. The data underlying this figure can be found in https://doi.org/10.6084/m9.figshare.28517873. **(c)** Penguins’ heading deviation from the line-of-sight direction generally increases in a non-linear fashion with respect to increasing dive depth and prey pursuit rate.
(TIF)

S12 Fig 3D scatter plot of angular heading differences among real penguin, line-of-sight, and ocean current directions. This figure illustrates the angular differences between the real penguin’s travel vector heading (relative to the water), the line-of-sight heading to the colony, and the ocean current heading. *X*-axis (red *x*–*y* projection): Angular difference between penguin heading and line-of-sight heading to the colony. *Y*-axis (green *z*–*x* projection): Angular difference between penguin heading and ocean current heading. *Z*-axis (blue *y*–*z* projection): Angular difference between line-of-sight heading to the colony and ocean current heading. Each red data point represents the mean angular difference calculated per 0.01 bin of each penguin’s proportion of distance travelled. The data underlying this figure can be found in https://doi.org/10.6084/m9.figshare.28517873.
(TIF)

S13 Fig Comparison of ocean current strength conditions during commuting and resting phases of penguins’ return journeys. Resting was defined as surface periods ≥60 s and commuting as all times when birds were at depth (diving). **(a)** shows the average current speed (m/s) during commuting and resting phases, while **(b)** depicts the differences in resultant speeds (post-current integration versus pre-current integration) for the two activity types. Boxplot (boxes encompass the 25% to 75% interquartile range, horizontal bars reflect the median and whiskers extend to 1.5 * Interquartile range) were constructed using the mean values per bird across activity types. **(c)** Presents an example of a penguin’s return journey exhibiting extended flow-assisted surface resting during slack current conditions. Red arrows denote the Actual bird headings and blue arrows denote the current direction. Arrow lengths are proportional to the speed of travel (both for the bird and the current). The data underlying this figure can be found in https://doi.org/10.6084/m9.figshare.28517873.
(TIF)

S14 Fig Example data showing changes in depth, prey pursuits, and heading distributions of mean circular headings per dive and dive phase for a single penguin. The top panel shows the number of prey pursuits (black bars) and depth profiles over time, with outbound dives indicated in blue and inbound dives in red. The bottom panel presents circular rose plots showing the mean headings per dive phase, color-coded as follows: descent (green), bottom phase (cyan), ascent (red), and surface/sub-surface (orange). It also includes the mean LoS heading towards the colony (black) and the ocean current heading (blue). Grand hourly means of these values are represented by arrows inside the circular plots. The data underlying this figure can be found in https://doi.org/10.6084/m9.figshare.28517873.
(TIF)

## Abbreviations

COT: cost of transport
DD: daily diary
DR: dead-reckoned
GAMs: generalized additive models
ROMS: regional ocean modelling system
