# Supplementary material for: Penguins exploit tidal currents for efficient navigation and opportunistic foraging
Source: PLoS Biol. 2025 Jul 17;23(7):e3002981. doi: 10.1371/journal.pbio.3002981 (PMC12327074; doi:10.1371/journal.pbio.3002981)
Supplement: S1 Text — (DOCX) [file pbio.3002981.s001.docx]

**Text S1**

*Numerical Ocean Current Model*

Ocean currents were simulated using the Regional Ocean Modelling System (ROMS) [1], which discretized the primitive equations vertically using stretched terrain-following coordinates over variable topography and horizontally using orthogonal curvilinear coordinates on a staggered Arakawa C-grid. Vertical mixing was parameterized using the Mellor and Yamada [2] scheme, and bathymetry was based on digitized nautical charts. The computational grid had three open boundaries (south, west, and north), where tidal amplitudes and phases of eight principal constituents (four semidiurnal: M₂, S₂, N₂, K₂, and four diurnal: K₁, O₁, P₁, Q₁), two long-term constituents (M_f_ and M_m_), and three higher harmonics (M₄, MS₄, and MN₄) were imposed, interpolated from the TPXO6 global tidal model [TPXO6, 3]. Further details on model setup, including grid, forcings, and boundary conditions, are provided by Tonini and Palma [4]. The model was validated using over 10 years of in-situ data from the region.

Instantaneous current outputs (3D fields) were subjected to harmonic analysis to extract the tidal constituents in both 3D and depth-averaged (2D) fields. The primary tidal components contributing to the greatest variations in spring and neap tides (M₂, S₂, and N₂) were extracted. Phase adjustments were applied using the global XTide model (https://flaterco.com). The U (east-west) and V (north-south) components of flow were assembled [5], representing the flow speeds along two orthogonal axes: U denotes the horizontal flow in the East-West direction, while V represents the flow in the North-South direction (Fig. S1). Currents were vertically averaged, as there was no significant variation from surface to bottom in the instantaneous currents within the San Lorenzo Gulf. The prevailing ocean currents relative to the penguins' line-of-sight toward the colony predominantly flowed either East-West or West-East, depending on the tidal phase (Fig. S2).

*Dead-Reckoning Speed Estimates*

Horizontal swimming speed was estimated using three approaches based on depth and body pitch:

1. ***Surface Swimming*:** When a bird was at a depth ≤ 0.3 m and absolute pitch angle (derived from the static surge acceleration [6]) < 10°, speed was set to 0.416 m/s (1.5 km/h), based on the surface swimming speed reported for the congeneric African penguin (*Spheniscus demersus*) [7].
2. ***Underwater Swimming with Low Body Pitch:*** For depths > 0.3 m and pitch angle < 10°, speed was set to 2.1 m/s, the average of two reported modal commuting speeds for Magellanic penguins: 2.0 m/s [8] and 2.2 m/s [9].
3. ***Underwater Swimming with High Body Pitch:*** Following Ropert-Coudert, Kato [10], when depth > 0.3 m and absolute pitch angle ≥ 10°, swimming speed (s) was estimated using:

$s= \frac{\Delta d}{\tan\left( \theta\bullet\frac{\pi}{180} \right)}$ (1)

where $s$ is in m/s, $\Delta d$ is the rate of depth change (m/s), and $\theta$ is the body pitch angle (= the angle of descent of ascent in the water column) in degrees. Speed values were capped at 5 m/s to avoid unrealistic estimates [11]. Note that in this study, we used horizontal speed in Equation (1) (main text) to maintain methodological consistency with Luna-Jorquera and Culik [12], whose metabolic cost equation was derived using speeds constrained to a horizontal plane. This approach also prevents compounding uncertainties associated with estimating 3D speed–power relationships, where air compression alters buoyancy and energy allocation depending on depth and swim angle [11]. Importantly, our dataset confirms that horizontal movement is the dominant component of swimming speed, with a median ratio of horizontal to total 3D speed of 0.989 (mean: 0.930), indicating that the primary mode of travel occurred in the horizontal dimension.

*GPS-Corrected Dead-Reckoning Procedure*

Pitch, roll, and heading were derived using the software Daily Diary Multi Trace (DDMT, <http://www.wildbytetechnologies.com> based on established methods Pedley [13], Bidder, Walker [14], and Gunner, Holton [6]. Subsequent analyses were conducted in R. Dead-reckoned (DR) tracks were corrected at approximately 1-minute epochs using a rolling time function. A GPS fix was retained only if the time interval since the previous retained fix was ≥60 seconds. After retaining a fix, the time counter reset, and the process continued. This method ensured that, during large gaps in GPS data (often due to penguins not remaining at the surface long enough for a fix), the next available fix was always included. To address the issue of GPS fixes being displaced in time (sometimes registering during subsequent dives), we reallocated the last GPS fix obtained during each inter-dive cycle (the period between surface intervals) to the last row index of the prior surface period. This prevented the correction of underwater movement paths based on surface positions. All other fixes registered when the bird was not at the surface were disregarded. Obvious outliers, identified through visual inspection, were initially removed.

To correct pressure drift in depth measurements, we calculated median pressure values when penguins were at or near the surface. These periods were identified by:

- **Pressure less than 10.4 mbar**: This is a high upper limit of potential drift for these study animals, as pressure recorded at the surface was typically ≥10 mbar.
- **Absolute rate of change of depth ≤ 0.05 m/s**: Surface differentials did not usually fluctuate above this threshold for this species.

Only median values from surface periods lasting ≥5 seconds were used. We then applied Trend Estimation with Asymmetric Least Squares (ALS) to these median values to determine the baseline drift over time, using the following parameters:

- Lambda (smoothing parameter): 0.001
- P (asymmetry parameter): 0.001
- Eps (numerical precision for convergence): 1e-8
- Maxit (maximum number of iterations): 50

This baseline was subtracted from the raw depth values over time. Any resultant depth values less than 0 m were set to 0 m. Additionally, median pitch values from the identified surface periods were pooled to calculate a grand median per bird, which was used as their estimated pitch offset applied to all pitch values. Logical corrections ensured that pitch remained within the -90° to +90° range.

**References**

1. Shchepetkin, A.F. and J.C. McWilliams, *The regional oceanic modeling system (ROMS): a split-explicit, free-surface, topography-following-coordinate oceanic model.* Ocean Modelling, 2005. **9**(4): p. 347-404.

2. Mellor, G.L. and T. Yamada, *Development of a turbulence closure model for geophysical fluid problems.* Reviews of Geophysics, 1982. **20**(4): p. 851-875.

3. Egbert, G.D., A.F. Bennett, and M.G.G. Foreman, *TOPEX/POSEIDON tides estimated using a global inverse model.* Journal of Geophysical Research: Oceans, 1994. **99**(C12): p. 24821-24852.

4. Tonini, M.H. and E.D. Palma, *Tidal dynamics on the North Patagonian Argentinean Gulfs.* Estuarine, Coastal and Shelf Science, 2017. **189**: p. 115-130.

5. Pisoni, J.P., et al., *Internal solitary waves from L-band SAR over the Argentine inner Patagonian shelf.* Remote Sensing Letters, 2020. **11**(6): p. 525-534.

6. Gunner, R.M., et al., *Dead-reckoning animal movements in R: a reappraisal using Gundog.Tracks.* Animal Biotelemetry, 2021. **9**(1): p. 23.

7. Wilson, R., *The Jackass Penguin (Spheniscus demersus) as a pelagic predator.* Marine Ecology Progress Series, 1985. **25**(3): p. 219-227.

8. Wilson, R.P., Y. Ropert-Coudert, and A. Kato, *Rush and grab strategies in foraging marine endotherms: the case for haste in penguins.* Animal Behaviour, 2002. **63**(1): p. 85-95.

9. Wilson, R.P., et al., *Antennae on transmitters on penguins: balancing energy budgets on the high wire.* Journal of Experimental biology, 2004. **207**(15): p. 2649-2662.

10. Ropert-Coudert, Y., et al., *Time/depth usage of Adélie penguins: an approach based on dive angles.* Polar Biology, 2001. **24**(6): p. 467-470.

11. Ainley, D.G. and R.P. Wilson, *The Aquatic World of Penguins: Biology of Fish-birds*. 2023: Springer.

12. Luna-Jorquera, G. and B.M. Culik, *Metabolic rates of swimming Humboldt penguins.* Marine Ecology Progress Series, 2000. **203**: p. 301-309.

13. Pedley, M., *eCompass-Build and Calibrate a Tilt-Compensating Electronic Compass.* Circuit Cellar-The Magazine For Computer Applications, 2012(265): p. 1−6.

14. Bidder, O.R., et al., *Step by step: reconstruction of terrestrial animal movement paths by dead-reckoning.* Movement Ecology, 2015. **3**(1): p. 23.
