# Supplementary material for: Penguins exploit tidal currents for efficient navigation and opportunistic foraging
Source: PLoS Biol. 2025 Jul 17;23(7):e3002981. doi: 10.1371/journal.pbio.3002981 (PMC12327074; doi:10.1371/journal.pbio.3002981)
Supplement: S2 Text — (DOCX) [file pbio.3002981.s002.docx]

**Text S2**

*Conversions Between U and V Components (m/s) and Heading (°) and Speed (m/s)*

The magnitude (horizontal speed) of a vector ($\vec{V_{s}}$) was calculated using:

$\vec{V_{s}}= \sqrt{\left( U^{2}+ V^{2} \right)}$ (2)

The direction (heading) of a vector $V_{H}$, ranging from 0° to 360° (towards the vector of travel), was estimated using:

$V_{H}=mod\left( 90- \left( artan 2\left( V,U \right) \times\frac{180}{\pi} \right), 360 \right)$ (3)

Note: "mod" refers to the modulo operator.

To convert estimates of speed ($\vec{V_{s}}$) and heading ($V_{H}$) (e.g., bird speed and heading) to U and V components (with **+U** representing currents towards the East and **+V** representing currents towards the North), we performed the following steps:

- Add 180° to every $V_{H}$ ​value: This accounts for the meteorological convention where U and V vectors originate from a specific direction.
- Adjust for circular range: Subtract 360° from any $V_{H}$​ values greater than 360° to maintain a range between 0° and 360°.
- Convert $V_{H}$​ to the -180° to +180° scale: Subtract 360° from $V_{H}$​ values greater than 180°

To convert VS and VH estimates (e.g. bird speed and heading estimates) to U and V components (m/s) (+U representing currents towards the East and +V currents towards the North), 180 was first added to every VH value (because meteorological convention traditionally states the direction from which U and V vectors originate). To ensure circular range was maintained between 0^o^ and 360^o^, 360 was subtracted from values > 360. Lastly, for the below formula, VH had to be converted to the -180^o^ to +180^o^ scale and so 360 was subsequently subtracted from values > 180^o^.

The U and V components were then calculated using:

$U= -\vec{V_{s}} \times\sin\left( V_{H} \bullet\frac{\pi}{180} \right)$ (4)

$V= -\vec{V_{s}} \times\cos\left( V_{H} \bullet\frac{\pi}{180} \right)$ (5)
