# Supplementary material for: Penguins exploit tidal currents for efficient navigation and opportunistic foraging
Source: PLoS Biol. 2025 Jul 17;23(7):e3002981. doi: 10.1371/journal.pbio.3002981 (PMC12327074; doi:10.1371/journal.pbio.3002981)
Supplement: S3 Text — (DOCX) [file pbio.3002981.s003.docx]

**Test S3**

*Statistical Methods Expanded*

*Data Binning and Normalization:*

To address unequal sample sizes between penguins, we appropriately binned variables of interest (in some cases using two-dimensional bins). When comparing frequency distributions (e.g., heading differences), counts per bin were calculated for each penguin individually using 5° or 10° bins spanning -180° to +180°. These counts were normalized by dividing by the total count for each penguin, converting counts to proportions and thus accounting for unequal sample sizes. We then averaged these per-penguin proportions across all penguins to obtain the grand mean relative frequency for each bin. When comparing variables as a function of the proportion of total or straight-line distance travelled during penguin return journeys (ranging from 0 to 1), we calculated the mean of the variable within each bin (e.g., bins of size 0.05 or 0.01) for each penguin. These per-penguin means were then averaged across penguins to obtain the grand mean.

*Bootstrapped Kolmogorov-Smirnov (KS) Test:*

We used a bootstrapped Kolmogorov-Smirnov (KS) test to assess whether the distributions of penguin heading differences relative to the line-of-sight heading differed significantly between outbound and inbound phases, regardless of current strength. Bootstrapping accounted for integer ties in the data and provided robust estimates of the KS statistic. To address unequal and large sample sizes and to account for intra-individual variability, we resampled data within each penguin and trip phase during each of the 1,000 bootstrap iterations. Pairwise KS tests were conducted to compare distributions across all combinations of current conditions and trip phases, with resampling applied within each condition for each iteration.

*Generalized Additive Models (GAMs):*

To investigate the complex relationships between environmental and behavioural factors influencing Magellanic penguins' navigation strategies, we employed Generalized Additive Models (GAMs) using the *mgcv()* package in R. GAMs were chosen for their flexibility in modeling non-linear relationships and accommodating both fixed and random effects.

1. *Ease of Transport and Proportion of Distance Travelled:*

We used GAMs to model the relationship between ease of transport and the proportion of distance travelled during the return leg of penguins’ foraging trips. Separate models were constructed for EOT calculated using:

1. The real penguin travel vector relative to the ground.
2. The fully-compensated travel vector relative to the ground.

These were assessed across two EOT variants:

1. Movement in any direction towards the colony.
2. Movement along the line-of-sight to the colony.

Each model included smooth terms for the proportion of distance travelled (in increments of 0.05) and accounted for interactions with the penguins' heading strategy (real vs. fully-compensated), specified using *bs = "fs"*. Random intercepts and slopes for penguin ID accounted for individual variation. Pairwise contrasts between heading strategies within distance bins were conducted to identify significant differences across distances. Mean values per bird, heading strategy, EOT variant, and distance bin were used in this GAM.

1. *Deviation of Real Penguin Travel Vector Headings (Relative to the Ground) from the Line-of-Sight Heading:*

To investigate the deviation of penguins’ real travel vector headings relative to the water from their intended line-of-sight directions toward the colony during return trips, we developed a GAM accounted for individual variability and non-linear effects of environmental and behavioural factors. The model incorporated random smooth effects to capture individual-specific differences across the proportion of distance travelled. Non-linear relationships were modelled for ocean current speed, angular deviation between penguin and ocean current headings, and differences in resultant travel speeds before and after current integration. Additionally, smooth terms were included to account for maximum dive depth and prey pursuit rate. A high-dimensional tensor product interaction assessed the interplay among ocean current speed, angular deviation between penguin and ocean current headings, and resultant travel speed difference. For each bird, mean or summed values (for prey pursuits) were calculated per 0.01 proportion of total distance travelled prior to modelling. The summed number of prey pursuits were divided by the total time spent underwater per epoch of distance travelled to convert to a standardised rate. Prior to modelling, data were aggregated by calculating mean or summed values for each penguin across increments of 1% of total distance travelled. The summed number of prey pursuits was standardized by dividing by the total time spent underwater per distance epoch, and angular deviations were transformed to their absolute values to emphasize the magnitude of deviation over directional information. Model selection was guided by residual diagnostics, assessments of concurvity, and evaluations of model fit.

To explore the interactions between key predictors in the GAM output, we utilized the *vis.gam* function to generate three-dimensional plots. These plots illustrate how the response variable—penguins' heading deviation from the line-of-sight direction—varies in relation to combinations of important predictors. Specifically, we examined the following interactions:

1. Angular Difference and Resultant Speed:

Predictors: The angular difference between the penguin's heading and the ocean current's heading, and the resultant speed difference after accounting for the current.

1. Angular Difference and Ocean Current speed:

Predictors: The angular difference between the penguin's heading and the ocean current's heading, and the ocean current speed.

1. Maximum Dive Depth and Prey Pursuit Rate:

Predictors: The maximum dive depth, and the prey pursuit rate.

In all cases, the current speed was held constant at its median value to isolate the effects of the other variables and provide a clearer understanding of their interactions with the response variable.
