# Supplementary material for: Penguins exploit tidal currents for efficient navigation and opportunistic foraging
Source: PLoS Biol. 2025 Jul 17;23(7):e3002981. doi: 10.1371/journal.pbio.3002981 (PMC12327074; doi:10.1371/journal.pbio.3002981)
Supplement: S1 Table — (i) The real penguin travel vector relative to the ground. (ii) The fully-compensated travel vector relative to the ground. (PDF) [file pbio.3002981.s004.pdf]

Table S1. Summary of GAM Results for Ease of Transport Models:
i. The real penguin travel vector relative to the ground.
ii. The fully-compensated travel vector relative to the ground.

Term	Model (i) (Any Direction)	Model (ii) (Line-of-Sight)
**Parametric Coefficients**		
**(Intercept)**	Estimate: 0.02940<br>Std. Error: 0.00122<br>t-value: 24.20<br>p-value: < 0.001 ***	Estimate: 0.02244<br>Std. Error: 0.00135<br>t-value: 16.66<br>p-value: < 0.001 ***
**Heading Strategy (fully-compensated vs. Real)**	Estimate: -0.00467<br>Std. Error: 0.00045<br>t-value: -10.36<br>p-value: < 0.001 ***	Estimate: 0.00228<br>Std. Error: 0.00045<br>t-value: 5.034<br>p-value: < 0.001 ***
**Approximate Significance of Smooth Terms**		
**s(Proportion of Distance Traveled): Real Strategy**	edf: 4.025<br>F-value: 15.273<br>p-value: < 0.001 ***	edf: 5.818<br>F-value: 6.810<br>p-value: < 0.001 ***
**s(Proportion of Distance Traveled): fully-compensated Strategy**	edf: 5.604<br>F-value: 3.016<br>p-value: 0.0034 **	edf: 5.580<br>F-value: 2.975<br>p-value: < 0.001 ***
**s(Penguin ID)**	edf: 19.275<br>F-value: 135.362<br>p-value: < 0.001 ***	edf: 20.967<br>F-value: 234.618<br>p-value: < 0.001 ***
**s(Proportion of Distance Traveled, Penguin ID)**	edf: 22.792<br>F-value: 170.796<br>p-value: < 0.001 ***	edf: 22.859<br>F-value: 264.214<br>p-value: < 0.001 ***
**Model Statistics**		
**Adjusted R-squared**	0.352	0.346
**Deviance Explained**	38.4%	38%
**Scale Estimate**	5.4909e-05	5.5455e-05
**Number of Observations (n)**	1080	1080

Significance Codes: *** $p < 0.001$; ** $p < 0.01$; * $p < 0.05$; . $p < 0.1$; $p \geq 0.1$ (not significant).
