# Supplementary material for: Penguins exploit tidal currents for efficient navigation and opportunistic foraging
Source: PLoS Biol. 2025 Jul 17;23(7):e3002981. doi: 10.1371/journal.pbio.3002981 (PMC12327074; doi:10.1371/journal.pbio.3002981)
Supplement: S2 Table — (PDF) [file pbio.3002981.s005.pdf]

Table S2. Summary of GAM Results for Deviation of Penguin Heading from Line-of-Sight Heading.

Term	Estimate	Std. Error	t-value	p-value
**(Intercept)**	2.816	0.081	34.925	< 0.001 ***

Smooth Term	edf	F-value	p-value
**s(Proportion of Distance, ID) (Random smooth for distance by penguin ID)**	223.34	15.557	< 0.001 ***
**s(Current Speed)**	4.08	1.28	< 0.001 ***
**s(Angular Difference between Penguin and Current Headings)**	5.67	2.15	< 0.001 ***
**s(Resultant.speed) (Resultant Speed after Current Integration)**	4.16	2.38	< 0.001 ***
**s(Maximum Dive Depth)**	2.37	2.39	<0.001 ***
**s(Prey Pursuit Rate)**	2.38	0.77	0.024 *
**Tensor Product Interaction of Angular Difference between Penguin and Current Headings, Current Speed, and Resultant Speed**	14.44	0.46	<0.001 ***

Model Statistics

- **Adjusted R-squared:** 0.704
- **Deviance Explained:** 57.4%
- **Scale Estimate:** 0.295
- **Number of Observations (n):** 2,697

Significance Codes: *** $p < 0.001$; ** $p < 0.01$; * $p < 0.05$; . $p < 0.1$; $p \geq 0.1$ (not significant).
