# Supplementary figures and images for: Penguins exploit tidal currents for efficient navigation and opportunistic foraging
Source: PLoS Biol. 2025 Jul 17;23(7):e3002981. doi: 10.1371/journal.pbio.3002981 (PMC12327074; doi:10.1371/journal.pbio.3002981)

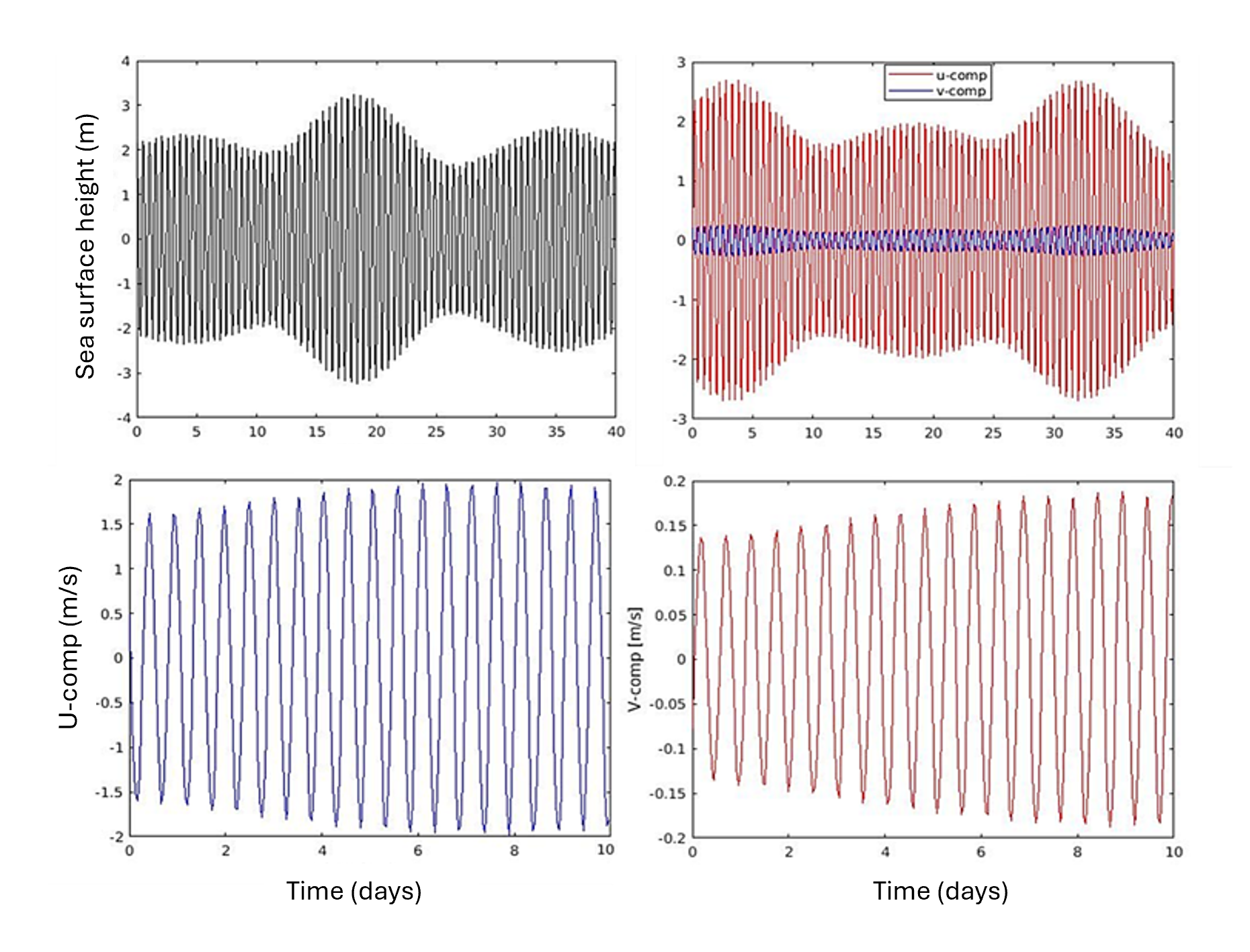

Supplement: S1 Fig — The top panel shows the variation in elevation and the U (east–west) and V (north–south) components of ocean currents in the study region over a 40-day period. The bottom panel presents a zoomed-in view of a 10-day section focusing on the phases of the U and V current components. The plot demonstrates periods dominated by cross-currents, particularly highlighting the U component’s influence on ocean dynamics. The data underlying this figure can be found in https://doi.org/10.6084/m9.figshare.28517873. (TIF) [file pbio.3002981.s006.tif]

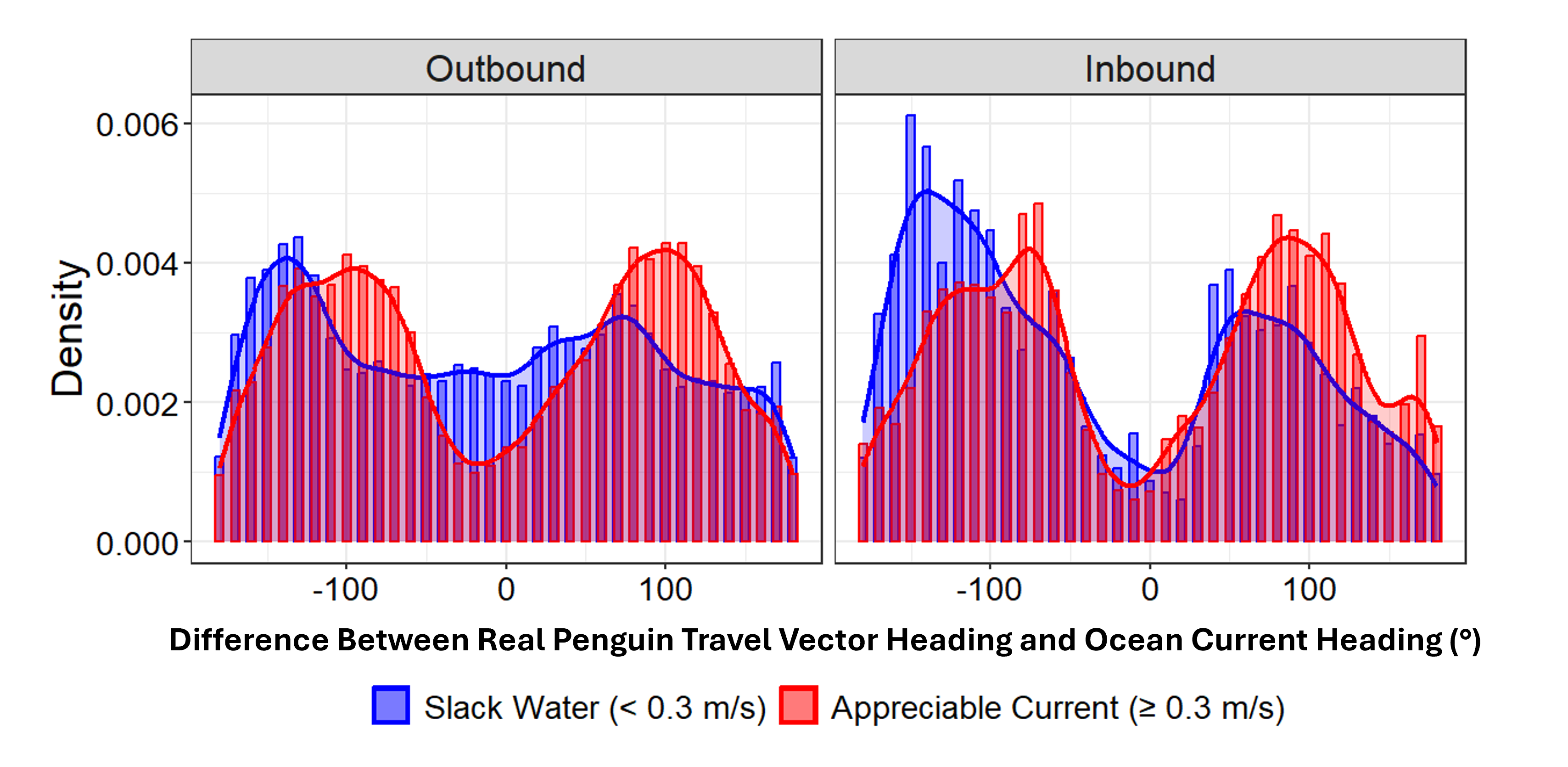

Supplement: S2 Fig — This plot shows the difference between the penguin’s travel vector heading (relative to the water) and the ocean current direction. The distribution is often bimodal, likely due to encounters with cross-currents. This bimodality is more pronounced during the return phase, suggesting more frequent cross-current navigation. The data underlying this figure can be found in https://doi.org/10.6084/m9.figshare.28517873. (TIF) [file pbio.3002981.s007.tif]

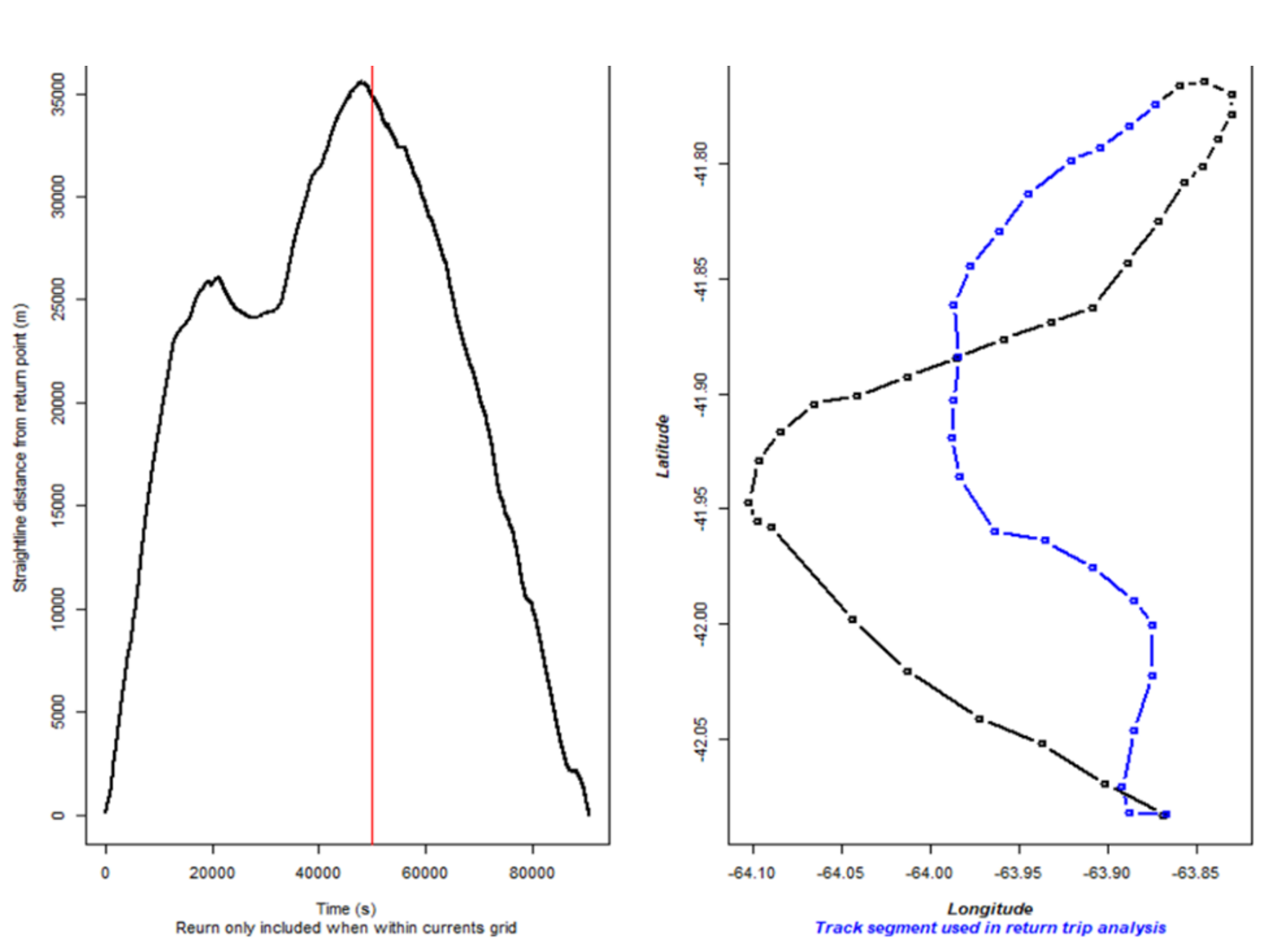

Supplement: S3 Fig — The left plot depicts the shortest straight-line distance from the penguin to the colony over time. The red vertical line indicates the start of the return phase, determined by selecting all data after the first GPS fix that occurred during the beginning of a continuous downward trend in straight-line distance to the colony. The right plot shows the penguin’s movement track (15-min GPS fix intervals shown). The blue section of the track represents the ‘Inbound’ phase, corresponding to the data after the red vertical line in the left plot. The data underlying this figure can be found in https://doi.org/10.6084/m9.figshare.28517873. (TIF) [file pbio.3002981.s008.tif]

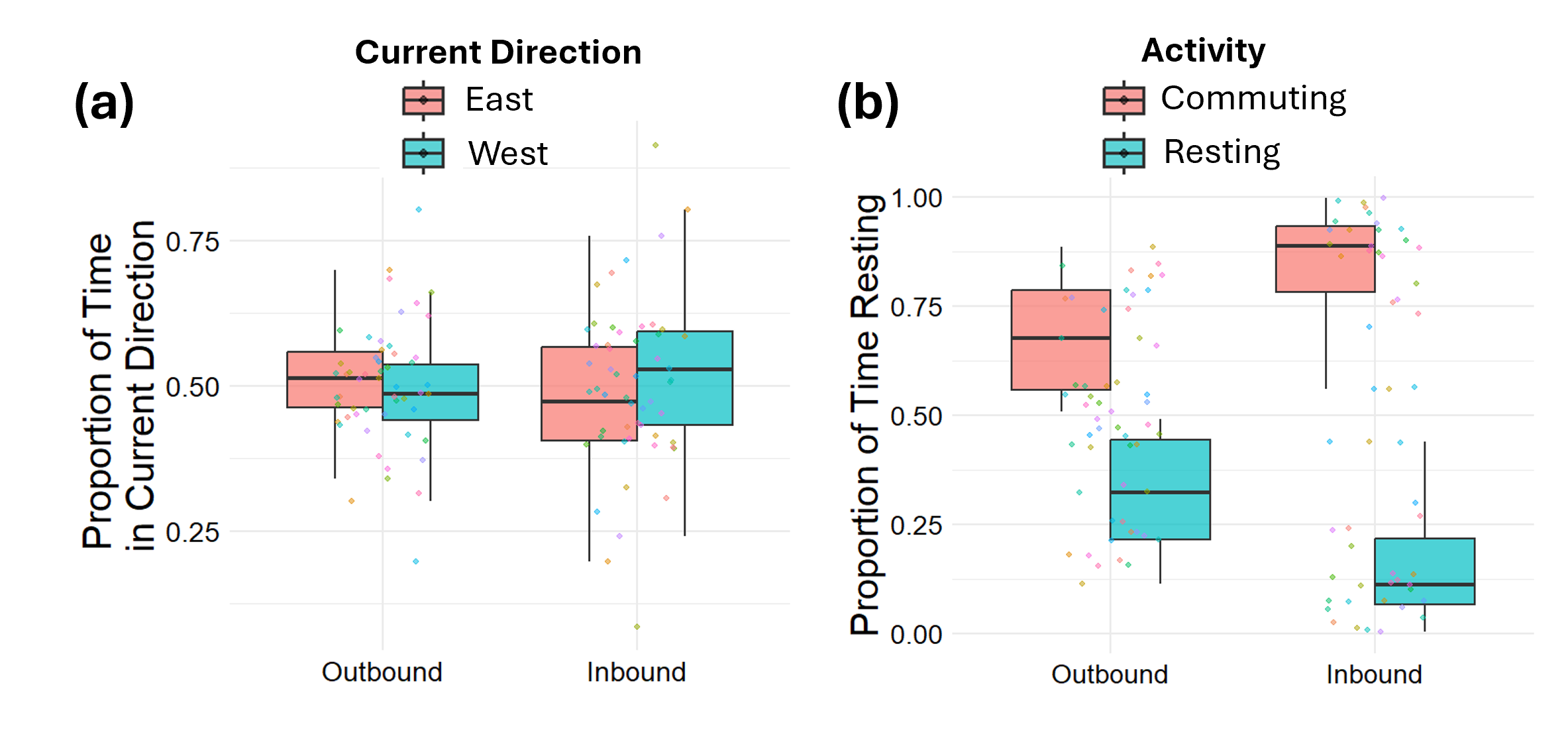

Supplement: S4 Fig — (a) Shows the proportion of time penguins experienced eastward-facing and westward-facing currents during outbound and inbound trips. For Inbound trips, the mean (±1 SD) current speed was 0.686 ± 0.243 m/s for eastward currents and 0.489 ± 0.183 m/s for westward currents; for Outbound trips, mean speeds were 0.628 ± 0.291 m/s (eastward) and 0.533 ± 0.178 m/s (westward). (b) Displays the proportion of time spent in each activity type (resting versus commuting) during outbound and inbound trips. Resting was defined as surface periods ≥60 s and commuting-specific to this figure-as all other times. Boxplot (boxes encompass the 25% to 75% interquartile range, horizontal bars reflect the median and whiskers extend to 1.5 * Interquartile range). Jittered points show each bird’s data point. The data underlying this figure can be found in https://doi.org/10.6084/m9.figshare.28517873. (TIF) [file pbio.3002981.s009.tif]

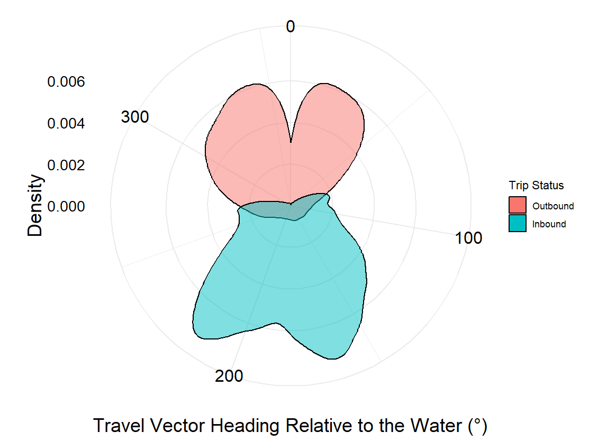

Supplement: S5 Fig — A circular density plot (polar coordinates) shows the absolute heading (0–360°, binned in degrees from North) for inbound (blue) versus outbound (red) travel phases. Both phases exhibit a broadly bi-modal distribution. The polar axis begins at 0° (North) at the top, increasing counterclockwise to 360°. Density is depicted radially, and overlapping areas indicate where inbound and outbound headings coincide. The data underlying this figure can be found in https://doi.org/10.6084/m9.figshare.28517873. (TIF) [file pbio.3002981.s010.tif]

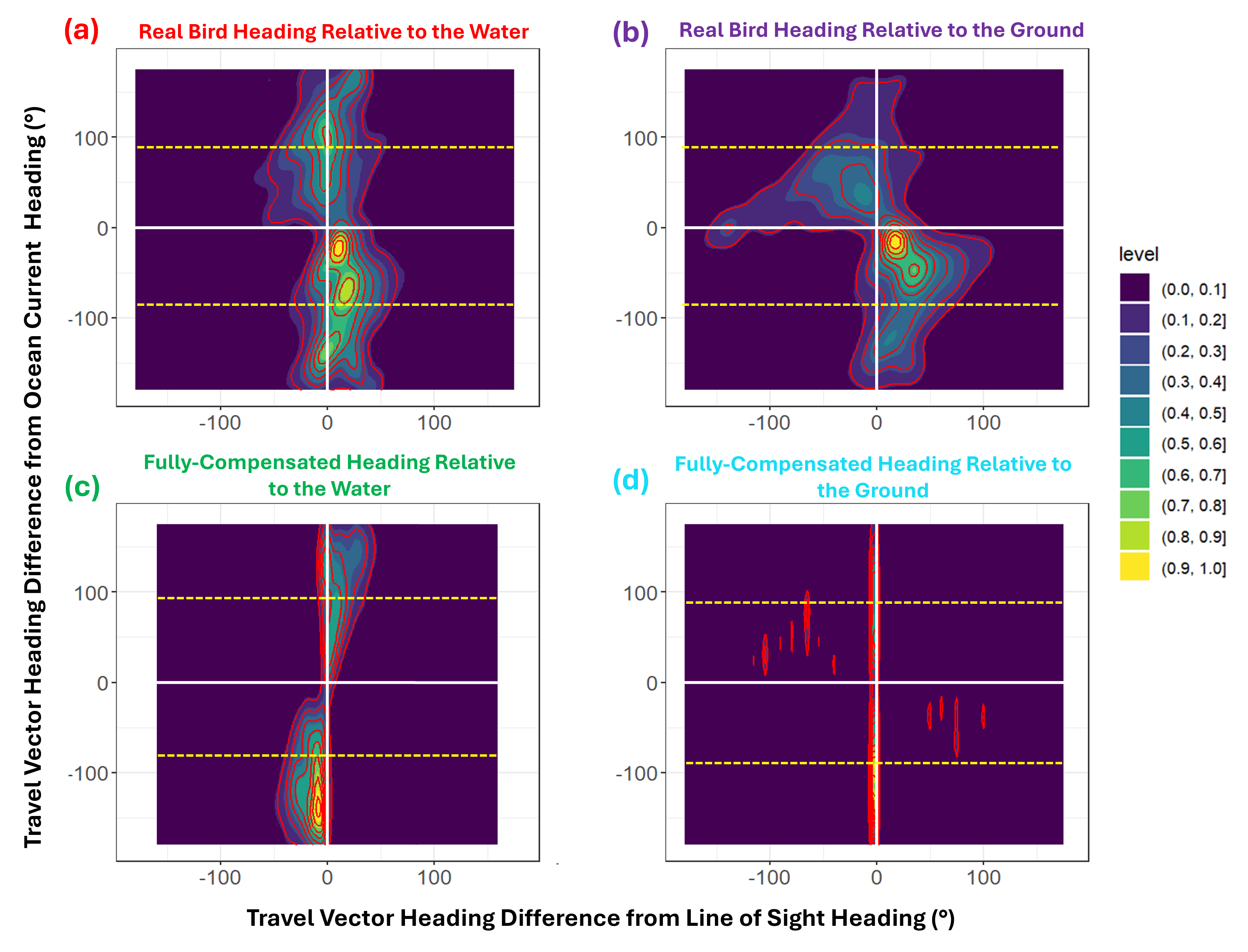

Supplement: S6 Fig — These contour plots compare real and theoretically fully-compensated penguin travel vectors relative to the line-of-sight heading to the colony (X-axis) and the ocean current direction (Y-axis). (a) Real penguin travel vector headings relative to the water generally align with line-of-sight trajectories toward the colony, though some skew is evident. (b) When accounting for ocean current effects, the resultant real penguin headings relative to the ground reveal current-assisted trends, roughly indicated within the dashed yellow lines. These headings show increased dispersion around the line-of-sight heading, with more occurrences of nearly opposing headings. (c) The theoretically fully-compensated penguin travel vector headings relative to the water, which ideally allow the birds to follow a direct line-of-sight path back to the colony after factoring in ocean current effects (as seen in panel d), would often require significant time swimming against the current, especially outside the dashed yellow lines. The data underlying this figure can be found in https://doi.org/10.6084/m9.figshare.28517873. (TIF) [file pbio.3002981.s011.tif]

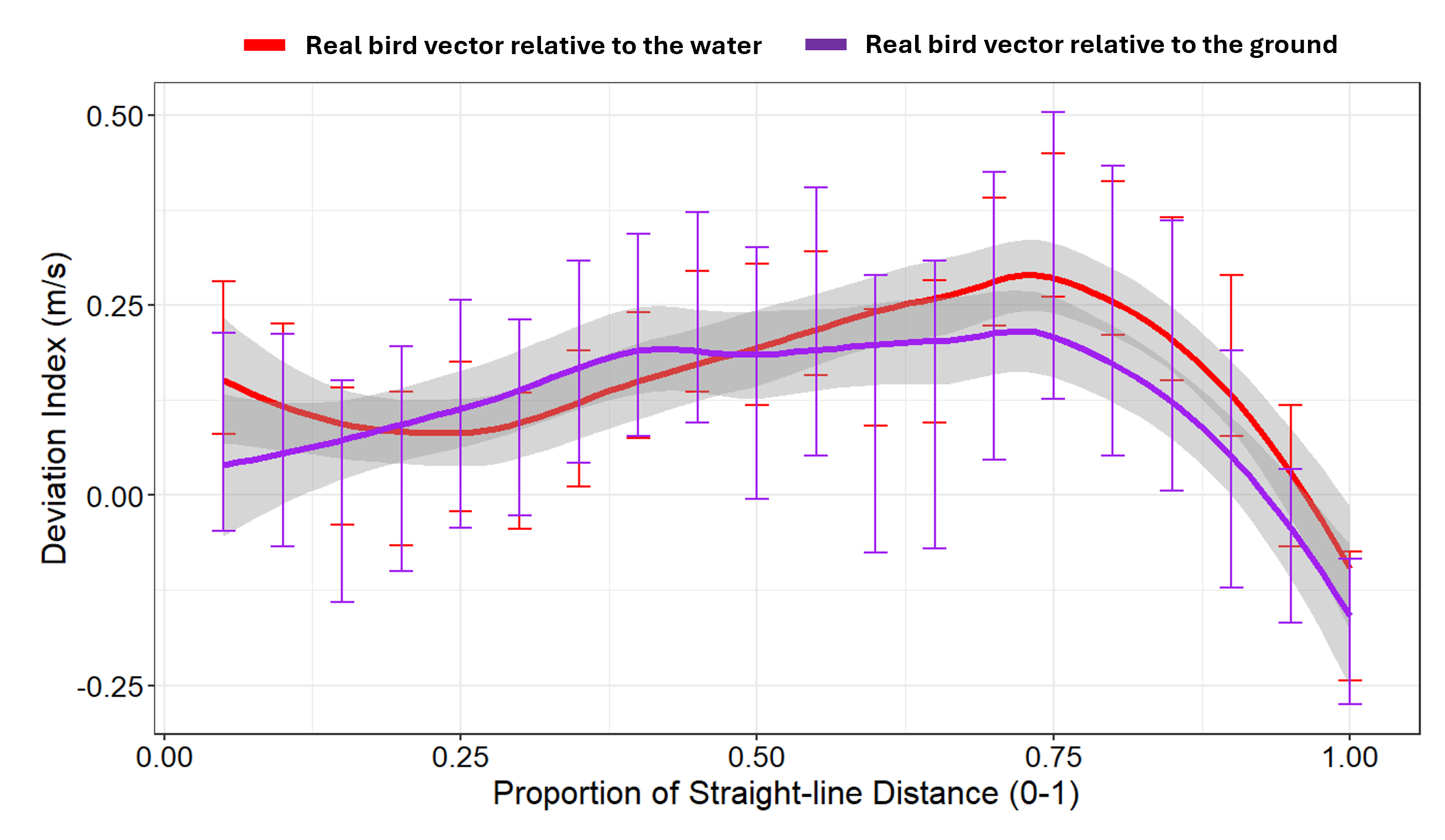

Supplement: S7 Fig — This index represents the lateral/off-axis component of each penguin’s travel relative to the direct line-of-sight heading. The red curve (mean ± SE) is computed using the bird’s original speed (before current integration) multiplied by sin(Δθ), where Δθ is the angular difference between the bird’s heading relative to the water and the line-of-sight. The purple curve (mean ± SE) uses the resultant (ground-based) speed and heading. Both are plotted against the proportion of straight-line distance (0–1) remaining in the return journey. The data underlying this figure can be found in https://doi.org/10.6084/m9.figshare.28517873. (TIF) [file pbio.3002981.s012.tif]

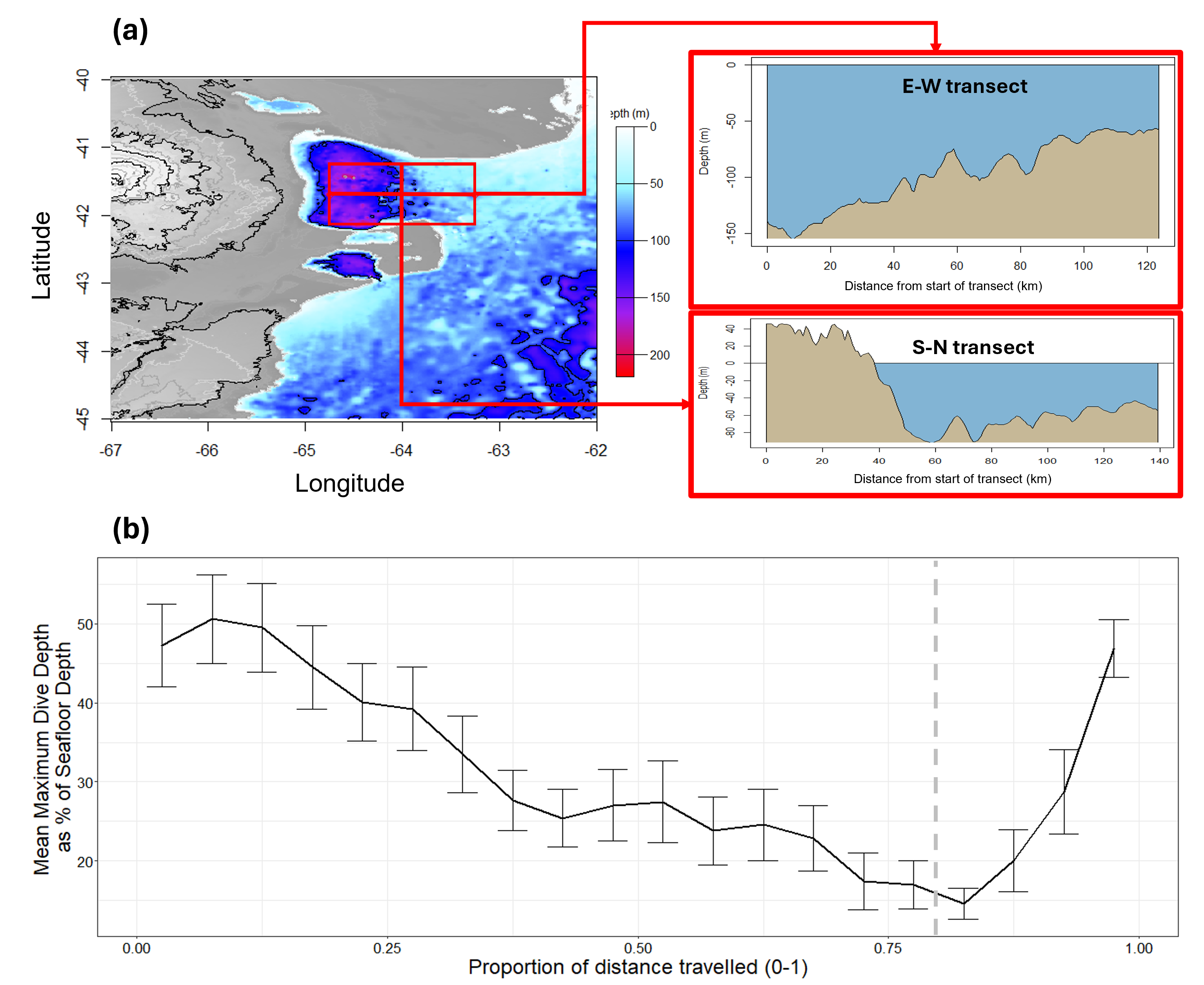

Supplement: S8 Fig — (a) Estimated seafloor depths in the region encompassing all penguin foraging journeys. (b) Mean maximum dive depth as a percentage of seafloor depth (±1 SE) across proportions of total distance travelled during return trajectories. Percentage values were averaged per 0.05 increments of total distance travelled. Bathymetric map derived from GEBCO_08 data. The data were obtained under GEBCO’s terms of use. Bathymetry is shown as a ‘bathy’ object in R using the marmap package [63]. Depth values (m) in (a) are plotted with a custom color ramp to highlight shallower shelf regions. The data underlying this figure can be found in https://doi.org/10.6084/m9.figshare.28517873. (TIF) [file pbio.3002981.s013.tif]

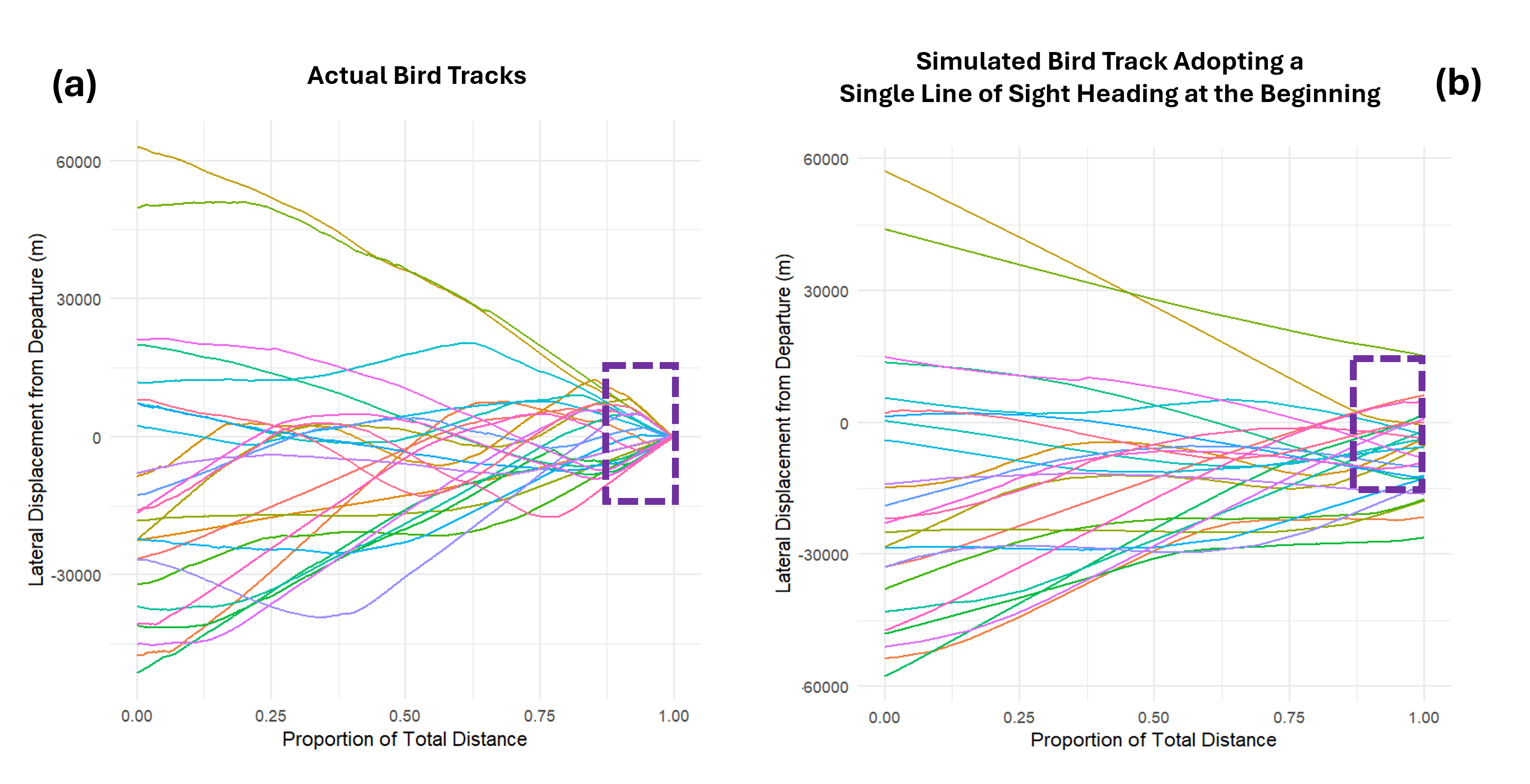

Supplement: S9 Fig — This figure illustrates the lateral displacement of penguins from their departure point at the colony during their return journeys. The y-axis represents lateral displacement (positive values indicate eastward movement; negative values indicate westward movement), and the x-axis represents the proportion of the total distance travelled (from 0 to 1). (a) Actual penguin data: Each line depicts an individual penguin’s lateral displacement over time. (b) Simulated scenario: Each penguin follows a fixed line-of-sight heading toward the colony without adjusting for current-induced drift effects. For both real and simulated penguins, starting coordinates were used as baselines to calculate absolute lateral displacement. The purple dashed boxes highlight displacement within 15 km of the colony during the final stages of the of the total distance travelled. The data underlying this figure can be found in https://doi.org/10.6084/m9.figshare.28517873. (TIF) [file pbio.3002981.s014.tif]

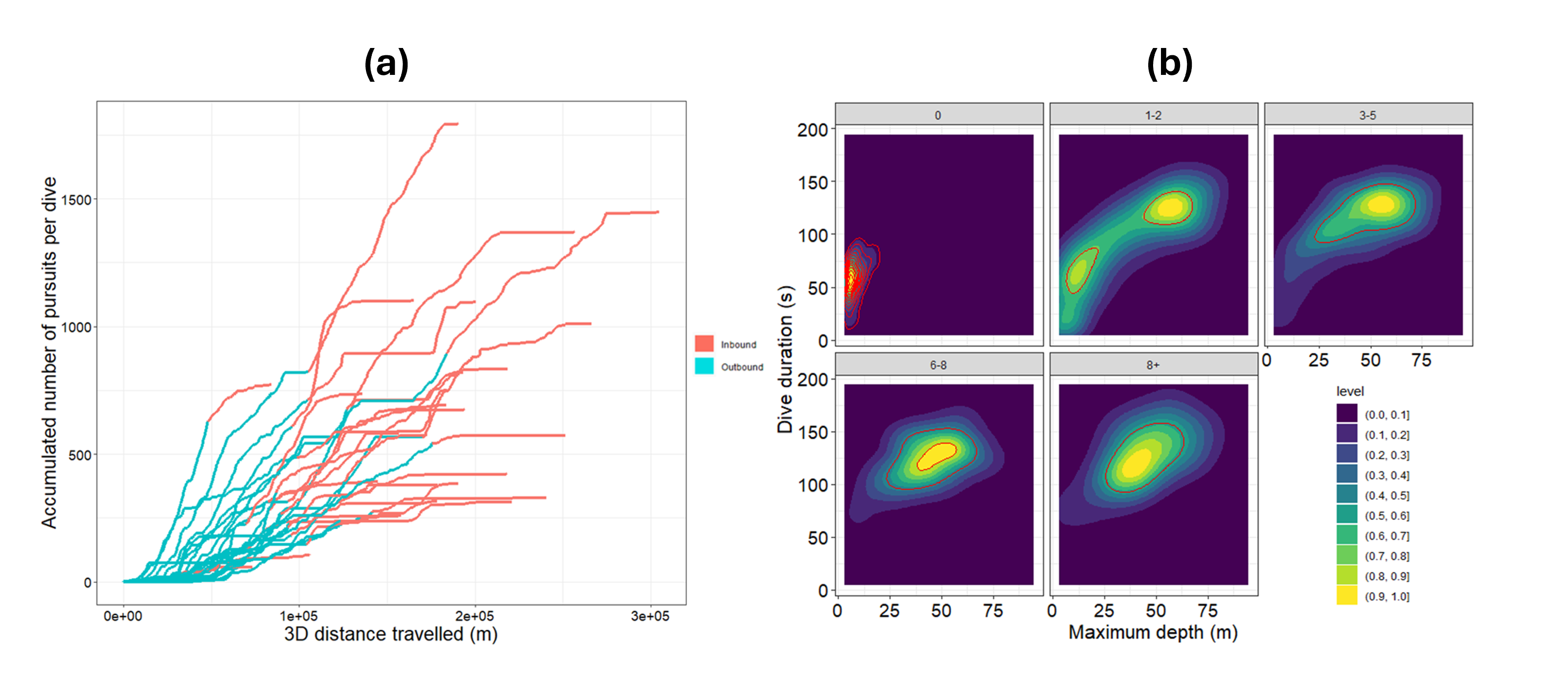

Supplement: S10 Fig — (a) The cumulative number of prey pursuits as a function of 3D distance travelled across each bird’s dead-reckoned path. Undulations in the dive profile were used as a proxy for prey pursuits, following methods outlined in Simeone and Wilson [37]. (b) The plots are faceted based on the number of prey pursuits per dive, illustrating how dive behaviour varies with foraging activity. The data underlying this figure can be found in https://doi.org/10.6084/m9.figshare.28517873. (TIF) [file pbio.3002981.s015.tif]

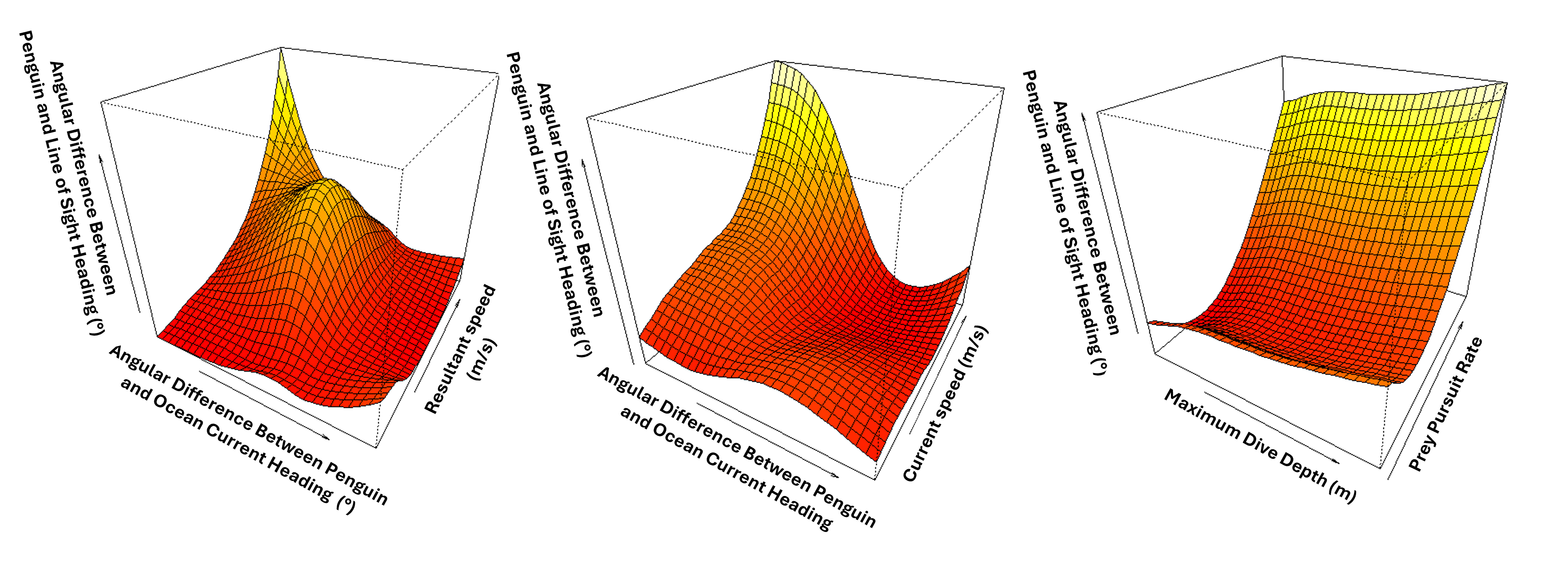

Supplement: S11 Fig — (a and b) Due to the nature of cross-currents in the region, penguins tend to align their movement with ocean current flow vectors. This alignment results in greater deviation from their line-of-sight trajectory, particularly at higher current speeds. However, this alignment also enables penguins to travel greater distances per unit time by increasing their resultant speeds. The data underlying this figure can be found in https://doi.org/10.6084/m9.figshare.28517873. (c) Penguins’ heading deviation from the line-of-sight direction generally increases in a non-linear fashion with respect to increasing dive depth and prey pursuit rate. (TIF) [file pbio.3002981.s016.tif]

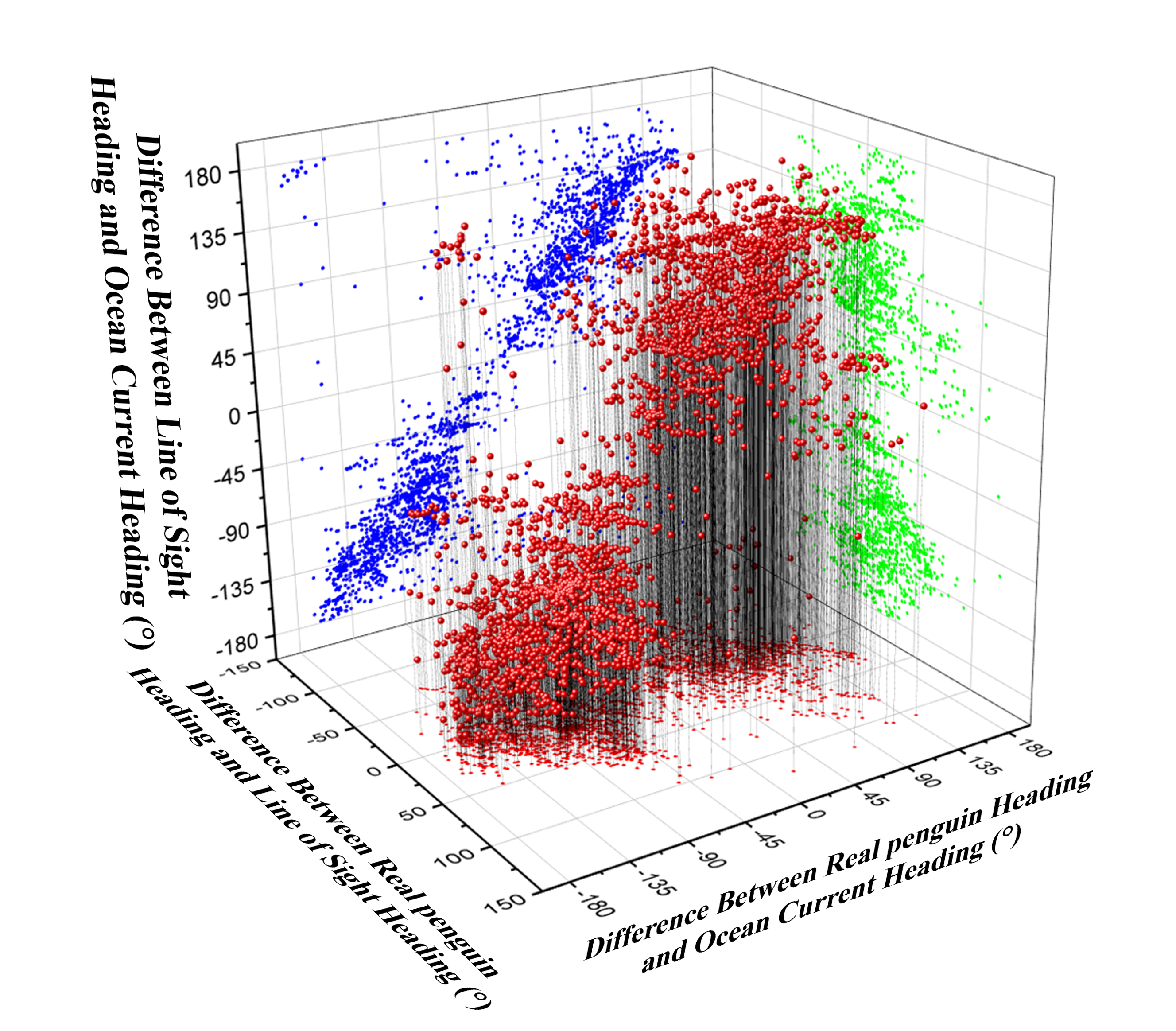

Supplement: S12 Fig — This figure illustrates the angular differences between the real penguin’s travel vector heading (relative to the water), the line-of-sight heading to the colony, and the ocean current heading. X-axis (red x–y projection): Angular difference between penguin heading and line-of-sight heading to the colony. Y-axis (green z–x projection): Angular difference between penguin heading and ocean current heading. Z-axis (blue y–z projection): Angular difference between line-of-sight heading to the colony and ocean current heading. Each red data point represents the mean angular difference calculated per 0.01 bin of each penguin’s proportion of distance travelled. The data underlying this figure can be found in https://doi.org/10.6084/m9.figshare.28517873. (TIF) [file pbio.3002981.s017.tif]

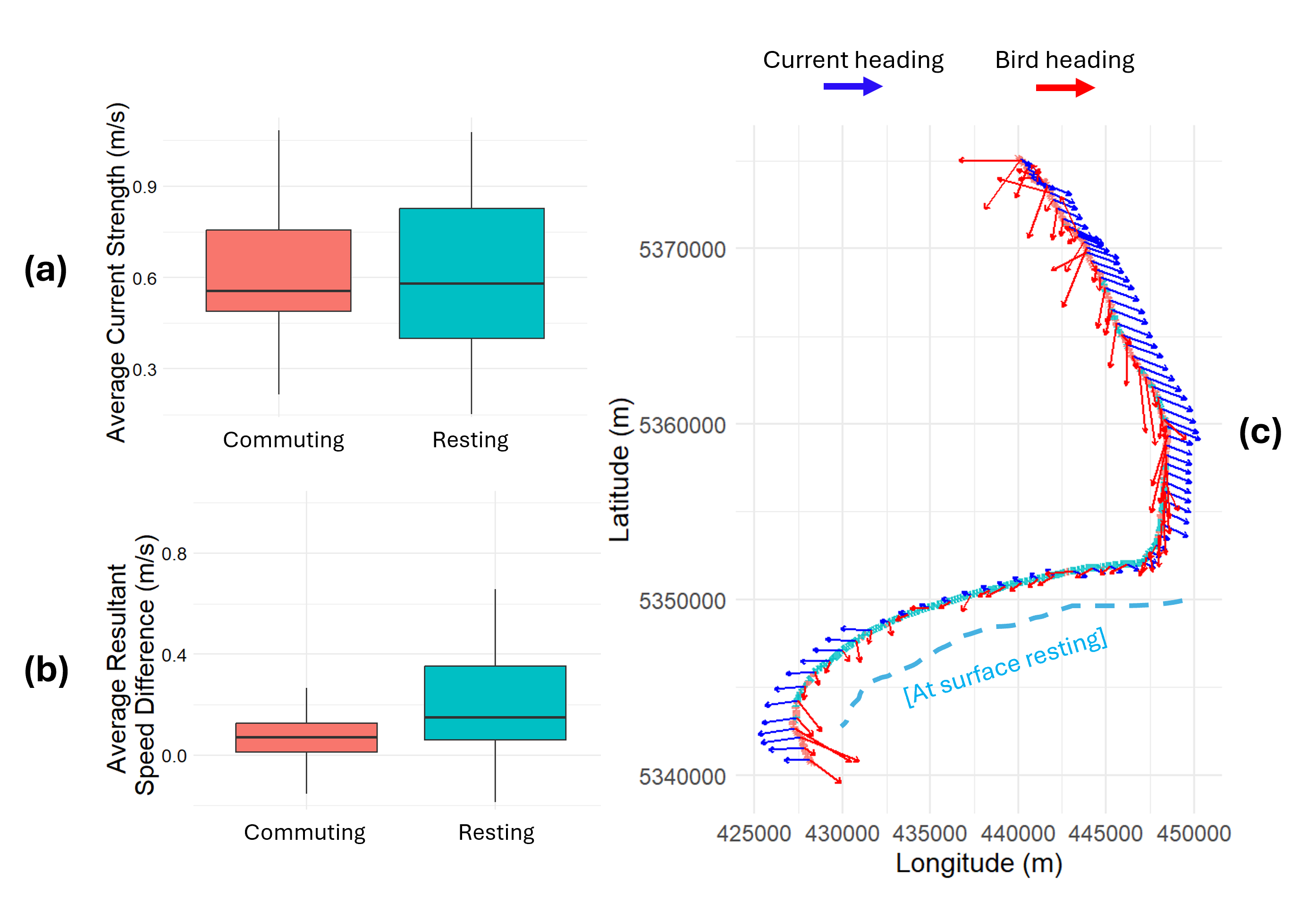

Supplement: S13 Fig — Resting was defined as surface periods ≥60 s and commuting as all times when birds were at depth (diving). (a) shows the average current speed (m/s) during commuting and resting phases, while (b) depicts the differences in resultant speeds (post-current integration versus pre-current integration) for the two activity types. Boxplot (boxes encompass the 25% to 75% interquartile range, horizontal bars reflect the median and whiskers extend to 1.5 * Interquartile range) were constructed using the mean values per bird across activity types. (c) Presents an example of a penguin’s return journey exhibiting extended flow-assisted surface resting during slack current conditions. Red arrows denote the Actual bird headings and blue arrows denote the current direction. Arrow lengths are proportional to the speed of travel (both for the bird and the current). The data underlying this figure can be found in https://doi.org/10.6084/m9.figshare.28517873. (TIF) [file pbio.3002981.s018.tif]

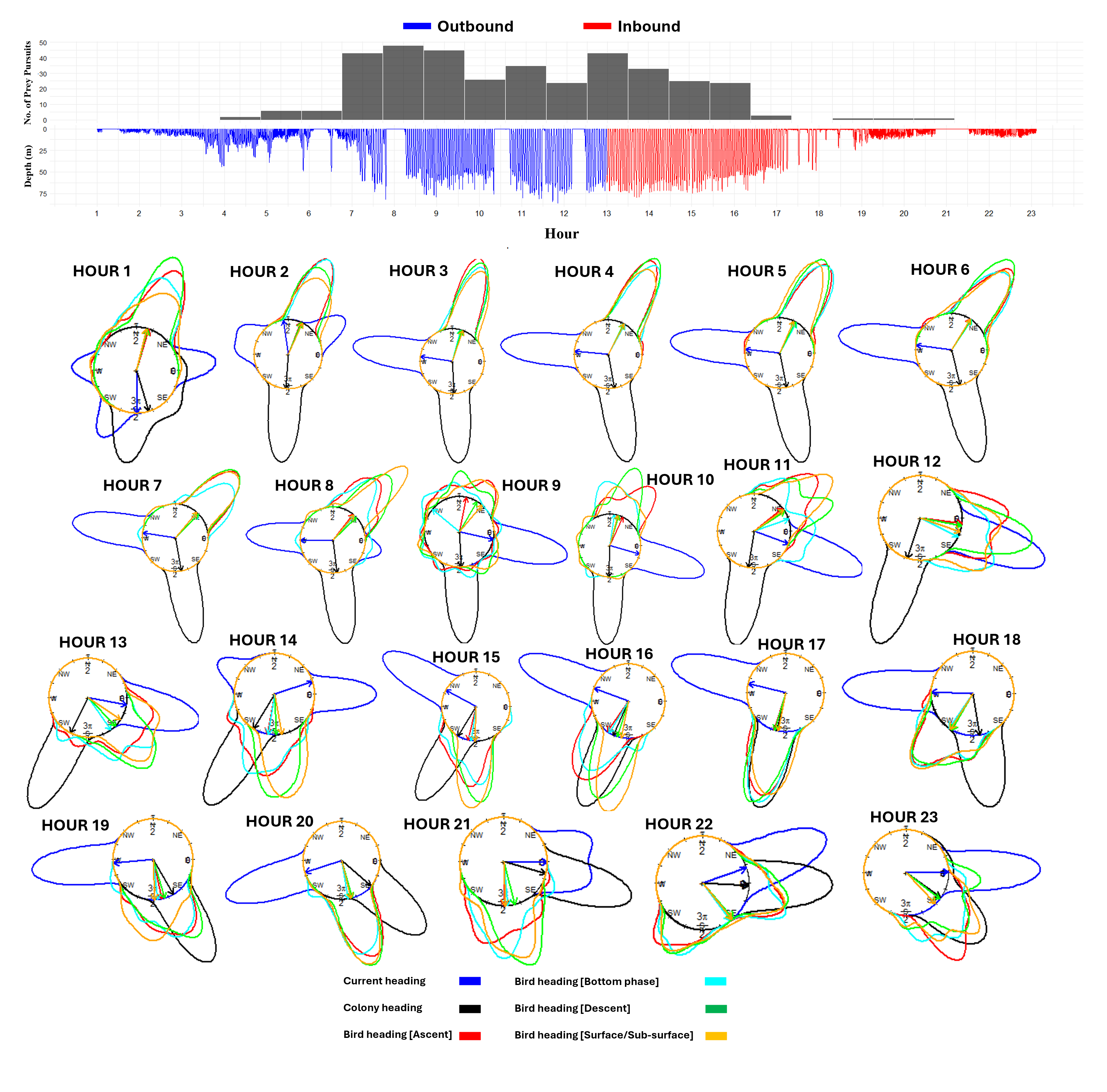

Supplement: S14 Fig — The top panel shows the number of prey pursuits (black bars) and depth profiles over time, with outbound dives indicated in blue and inbound dives in red. The bottom panel presents circular rose plots showing the mean headings per dive phase, color-coded as follows: descent (green), bottom phase (cyan), ascent (red), and surface/sub-surface (orange). It also includes the mean LoS heading towards the colony (black) and the ocean current heading (blue). Grand hourly means of these values are represented by arrows inside the circular plots. The data underlying this figure can be found in https://doi.org/10.6084/m9.figshare.28517873. (TIF) [file pbio.3002981.s019.tif]
